# Geometrically Optimized FDM-Printed Conductive TPU Bend Sensors for Hand Rehabilitation

**DOI:** 10.3390/s26082309

**Published:** 2026-04-09

**Authors:** Ahmet Özkurt, Damla Gürkan Kuntalp, Ozan Kayacan, Özlem Kayacan, Selnur Narin Aral

**Affiliations:** 1 Department of Electrical and Electronics Engineering, Dokuz Eylül University, İzmir 35390, Turkey; damla.kuntalp@deu.edu.tr; 2 Department of Textile Engineering, Dokuz Eylül University, İzmir 35390, Turkey; ozan.kayacan@deu.edu.tr (O.K.); ozlem.bicer@deu.edu.tr (Ö.K.); 3 Faculty of Physical Therapy and Rehabilitation, Dokuz Eylül University, İzmir 35330, Turkey; selnur.osun@deu.edu.tr

**Keywords:** bend sensor, 3D printing, conductive TPU, negative gauge factor, rehabilitation glove, wearable sensors, resistive

## Abstract

Flexible resistive bend sensors are essential for monitoring human movement in smart rehabilitation and soft robotics. However, widespread adoption is currently hindered by a trade-off between the high cost of metal-film technologies and the performance degradation (significant hysteresis and non-linearity) of low-cost carbon/polymer composites. This study presents a geometrically customizable bending sensor fabricated from conductive thermoplastic polyurethane (TPU) using Fused Deposition Modeling (FDM) technology as an accessible alternative to commercial sensors. By parametrically optimizing physical dimensions—including trace width, layer thickness, and pattern geometry—the sensors were tailored to achieve target resistance values within a target window of 20–50 kΩ (achieved: ~44 kΩ nominal) for specific finger-joint applications. Electromechanical characterization revealed a negative gauge factor (GF), where resistance decreases upon bending or elongation due to conductive pathway formation and densification within the polymer matrix. This behavior cannot affect sensor operation, and required bend-resistance responses were acquired using geometrical optimization. To compensate for inherent viscoelastic-induced hysteresis and non-linear behavior, a third-degree polynomial modeling approach was implemented. This modeling approach yielded a coefficient of determination (*R*^2^) of approximately 0.90. Compared to standard commercial sensors, the proposed FDM-printed design successfully overcomes geometric limitations while offering a cost-effective, high-performance solution for tailor-made wearable technologies and smart rehabilitation gloves.

## 1. Introduction

The rapid convergence of flexible electronics, soft robotics, and human–machine interfaces (HMIs) is reshaping healthcare monitoring and physical rehabilitation at an unprecedented pace. Market analyses project the global printed and flexible sensor sector will exceed USD 8 billion by 2028, driven primarily by demand in wearable health monitoring and prosthetic control applications [1]. At the clinical level, the ability to capture and quantify multi-joint kinematics in real time is central to evidence-based rehabilitation of neurological and musculoskeletal conditions, including post-stroke hemiplegia, rheumatoid arthritis, and spinal cord injury. Among the sensing modalities under investigation, flexible piezoresistive bend sensors occupy a strategically important position: they transduce mechanical deformation directly into an electrical signal, require no optical alignment, and are compatible with low-power microcontroller acquisition systems—attributes that make them well-suited for ambulatory, patient-worn monitoring and for immersive virtual reality (VR) data gloves used in therapeutic training environments [2,3].

A substantial body of literature has explored materials and fabrication strategies for flexible strain and bend sensing. Established approaches include mold casting with elastomeric composites (e.g., carbon black or carbon nanotube (CNT) fillers dispersed in polydimethylsiloxane (PDMS) or Ecoflex matrices), screen and inkjet printing of conductive inks onto polymer substrates, and laser-induced graphene (LIG) patterned on polyimide films [3,4,5]. These methods have demonstrated impressive performance metrics—Gauge Factors (GFs) exceeding 100 in nano-structured composites, sub-millisecond response times, and stable cyclic performance over thousands of deformation cycles. However, each approach carries inherent manufacturing constraints that impede widespread clinical adoption. Mold casting and solution-based processing require multi-step procedures, clean room access, or custom tooling that is impractical in rehabilitation equipment workshops. Screen printing, while scalable, produces sensors with fixed, predefined geometries that cannot be rapidly reconfigured for individual patient anatomy. LIG methods, although rapid, are substrate-limited and produce mechanically fragile sensing layers prone to delamination under repetitive large-strain deformation [5]. Commercial off-the-shelf bend sensors (e.g., SpectraSymbol resistive flex sensors) offer excellent linearity and repeatability but are manufactured in fixed standard dimensions that cannot be adapted to specific joint sizes—a critical limitation when sensor length must be precisely matched to the proximal interphalangeal (PIP) joint of a child or to the metacarpophalangeal (MCP) joint of a prosthetic hand [6]. More recently, wearable composite sensors have further raised the performance benchmark against which FDM-printed designs must be judged: aerogel–nanofiber hybrid architectures integrating simultaneous magnetism, strain, and pressure transduction have demonstrated real-time information encoding and gesture recognition [7], while hydrogel-sponge platforms coupled with deep-learning classification pipelines have achieved discriminable gesture language recognition from a single sensing element with accuracy exceeding 95% across multiple gesture classes [8]—underscoring the accelerating convergence of advanced composite materials and machine learning in flexible sensing systems.

Additive manufacturing, specifically Fused Deposition Modeling (FDM)—also termed Fused Filament Fabrication (FFF)—has emerged as a compelling alternative that directly addresses these geometric and accessibility limitations. By encoding sensor geometry in a digital design file and executing production on a desktop 3D printer costing less than USD 500, FDM enables rapid, geometry-agnostic sensor prototyping without specialized tooling. The commercial availability of conductive thermoplastic polyurethane (c-TPU) filaments—combining the high compliance (Shore 40A–92A hardness) of TPU with the electrical conductivity of dispersed carbon-black (CB) or graphitic filler networks—has unlocked the capacity to print flexible piezoresistive structures in a single manufacturing step [9,10]. The direct mechanical-to-electrical transduction of FDM-printed c-TPU sensors offers a significant practical advantage over alternative magnetic sensing modalities. While technologies such as magnetic tunnel junctions (MTJs) utilizing vortex-state anisotropy [11] and magnetic levitation platforms [12] provide high sensitivity and specialized characterization, they are constrained by the need for external magnetic infrastructure and rigid substrates. These requirements fundamentally prevent the rapid, patient-specific geometric customization that is uniquely afforded by FDM printing. Crucially, FDM’s parametric control over trace geometry (path width, layer height, infill density, and pattern topology) provides a direct lever for tuning nominal sensor resistance and electromechanical sensitivity without requiring any change in feedstock material. Recent work has confirmed that print resolution alone can shift the gauge factor of a c-TPU structure across nearly an order of magnitude, demonstrating the depth of design freedom available to sensor engineers [13]. This manufacturability advantage—the ability to produce a custom-dimensioned, electrically functional bend sensor within 30–60 min from concept to prototype—constitutes a qualitative shift relative to all prior fabrication paradigms.

Despite growing interest in FDM-printed flexible sensors, the published literature reveals several persistent gaps that limit the transition from demonstrator to deployable clinical device. First, the majority of studies focus on the chemical synthesis or composite formulation of novel conductive feedstocks, rather than on the systematic geometric optimization of commercially available filaments for target application constraints [14,15,16,17]. Second, the influence of key FDM process parameters—nozzle temperature, print speed, layer height, and infill pattern—on the resulting piezoresistive behavior of c-TPU structures has not been characterized in a unified parametric study that maps process inputs to measurable sensor outputs (nominal resistance, gauge actor, and hysteresis). Third, a phenomenon that is theoretically predicted for carbon-black-loaded elastomeric composites—the negative gauge actor (nGF), in which electrical resistance decreases upon mechanical deformation rather than following the positive piezoresistive trend of metallic gauges—has been reported anecdotally in FDM-printed sensors but has not been rigorously analyzed through the lens of percolation theory and quantum tunneling transport [10,14]. Without this physical mechanistic framework, nGF observations risk being dismissed by reviewers as measurement artifacts rather than recognized as an intrinsic and exploitable property of the composite microstructure. Fourth, while hysteresis arising from viscoelastic relaxation of the TPU matrix is widely acknowledged as a limiting factor for dynamic accuracy, most FDM sensor studies address it only qualitatively or apply simple linear correction factors. The more rigorous phenomenological frameworks—such as Prandtl–Ishlinskii (PI) operators or the Bouc–Wen model—that would enable real-time hysteresis compensation on embedded microcontrollers remain largely unexplored in the context of printed c-TPU sensors. Finally, few studies benchmark FDM-printed c-TPU sensors directly and quantitatively against commercial reference standards, which obscures their true competitive position in application-relevant performance metrics. Recent advancements in single-sensor machine learning (ML) systems, such as hydrogel-sponge composites [8] and multimodal aerogel–nanofiber architectures [7], have demonstrated that a single compliant transducer can support robust gesture recognition and real-time encoding. However, the analogous capacity of FDM-printed c-TPU sensors to function as the sole input for an embedded classifier has not yet been systematically evaluated. Consequently, their potential as low-cost, training-efficient inputs for ML-augmented rehabilitation workflows remains uncharacterized [8].

The present study addresses these gaps through a focused investigation into the design, fabrication, and electromechanical characterization of geometrically customizable, low-cost FDM-printed bend sensors based on a commercially available conductive TPU filament (Recreus Conductive Filaflex), targeting the constraints of hand rehabilitation instrumentation and VR data gloves. The principal contributions of this work are as follows. (i) Parametric geometric optimization: Sensor physical dimensions—including trace width, layer thickness, and U-shaped path topology—are systematically adjusted to achieve a target nominal resistance of approximately 20 kΩ suitable for direct interface with standard signal acquisition circuits, without any change in feedstock material. (ii) Process parameter rationale: Critical FDM printing parameters (nozzle temperature, infill density, layer height, extrusion width, and print speed) are explicitly reported and theoretically justified in terms of their effects on interlayer diffusion, conductive network formation, and mechanical integrity. (iii) Physical interpretation of the negative gauge factor: The observed nGF (GF = −1.33) is explained through a combined Poisson-effect transverse compression and percolation-pathway densification mechanism, situating the result firmly within the established physics of conductive polymer composites rather than treating it as an anomaly. (iv) Non-linear hysteresis modeling: A third-degree polynomial calibration model is applied to achieve R^2^ = 0.90 angle prediction reliability, and the path toward advanced Prandtl–Ishlinskii dynamic compensation is discussed as a prioritized future direction. (v) Comparative benchmarking: The fabricated sensors are compared directly to a commercial SpectraSymbol reference sensor and to selected state-of-the-art flexible sensor studies, providing a transparent assessment of competitive advantages and limitations. Together, these contributions establish that FDM-printed c-TPU sensors, when geometrically and electrically optimized through a structured parametric workflow, represent a viable, low-cost alternative to fixed-geometry commercial sensors for patient-customized rehabilitation technology [18,19,20].

Against this background, the key distinction of the present work can be stated explicitly. Prior FDM-printed c-TPU sensor studies have (i) characterised fixed-geometry structures without demonstrating systematic resistance targeting [9,10,14], (ii) reported the nGF as an observed anomaly without providing a physical mechanism [10,14], and (iii) omitted direct quantitative benchmarking against commercial references. The present study addresses all three gaps simultaneously in a single unified platform, using only commercially available filament and desktop equipment—a combination not previously reported in the flexible sensor literature.

## 2. Materials and Methods

### 2.1. Material Selection and Characterization

The fabrication of sensor bodies and integrated conductive pathways utilized two commercially available 1.75 mm diameter conductive thermoplastic polyurethane (c-TPU) filaments: Recreus Conductive Filaflex (Recreus Industries S.L., Elda, Spain) and RepRapper Electrically Conductive Filament (RepRapper Tech Co., Ltd., Hong Kong, China). Because neither manufacturer provided complete piezoresistive characterization data sufficient for sensor design, systematic preliminary electrical characterization was performed on both filaments prior to 3D printing.

**Figure 1:**
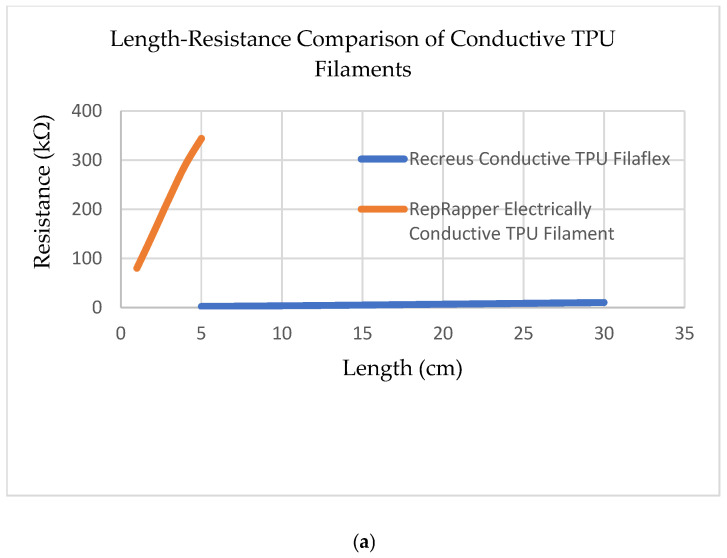

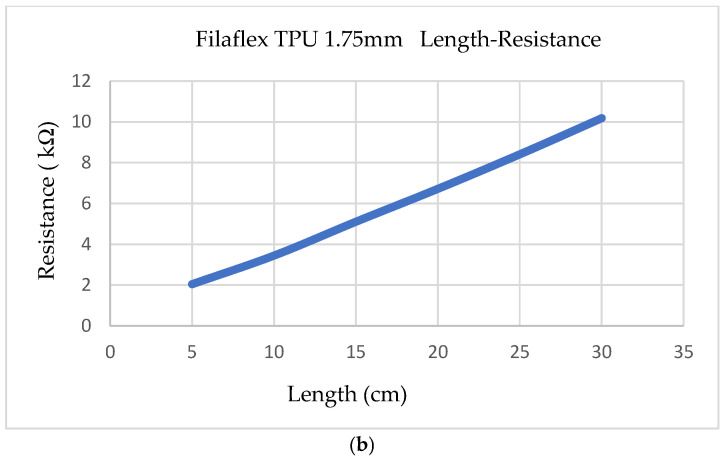
(**a**) Resistance Responses of two alternative conductive TPU filaments, (**b**) Detailed length-resistance response of chosen filament Recreus Conductive Filaflex.

Recreus Conductive Filaflex is characterized by a Shore 92A hardness and incorporates carbon black (CB) as the conductive filler phase, yielding a volumetric resistivity of approximately 3.9 Ω·cm—a value that situates it well above the percolation threshold and within the impedance range accessible to a standard Arduino Nano 10-bit analog-to-digital converter (ADC) operating at 5 V with a 47 kΩ reference resistor [21]. The RepRapper filament, in contrast, exhibited resistances exceeding 1 MΩ for trace lengths relevant to finger-joint sensing, rendering it incompatible with the target signal acquisition range. Two mechanisms explain this behavior within the framework of percolation theory [9]. First, the CB filler concentration in the RepRapper formulation is likely critically close to—or marginally below—the percolation threshold: the composition at which the conductive particle network undergoes an abrupt insulator-to-conductor transition. Near this threshold, small spatial variations in filler dispersion produce extreme resistance non-uniformity across the printed volume. Second, the thermal history experienced during FDM extrusion and rapid quench-cooling may have inhibited cohesive interparticle contact network formation, preventing CB agglomerates from redistributing into continuous conductive pathways. This is evidenced by the observation that RepRapper resistance increased further post-3D printing—a behavior consistent with the disruption of marginally established percolation pathways during the FDM re-melt and rapid cooling cycle [9]. Recreus Conductive Filaflex was therefore selected as the sole sensing material for all subsequent experiments. The information of the materials tested are given below as Table 1. Resistance Responses of two alternative conductive TPU filaments and detailed length-resistance response of chosen filament Recreus Conductive Filaflex are shown in Figure 1.

**Figure 2:**
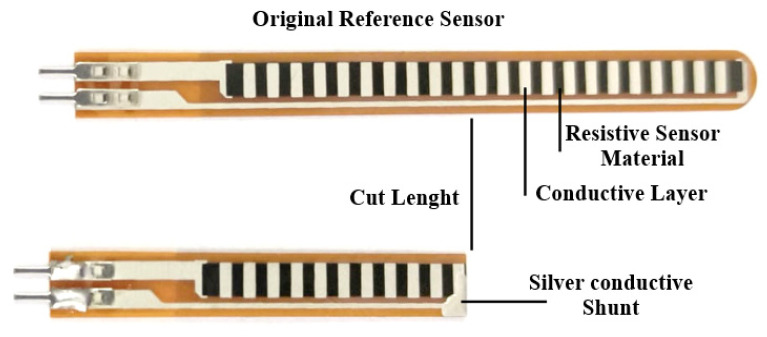
The reference commercial bend sensor from SpectraSymbol, Salt Lake City, UT, USA.

A commercial resistive bend sensor (SpectraSymbol, Salt Lake City, UT, USA) served as the performance benchmark throughout this study. It exhibits a near-ideal linear resistance–angle response (25–100 kΩ range, R^2^ = 0.99) but is available only in fixed standard lengths (44.5 mm and 73.7 mm), which prevents direct adaptation to arbitrary patient joint geometries—the central limitation motivating the present work [6]. In Figure 2, the reference commercial bend sensor from SpectraSymbol is shown.

### 2.2. FDM Fabrication and Print Parameter Optimization

Sensor prototypes were fabricated on a Bambu Lab P2S 3D printer (Bambu Lab Co., Ltd., Shenzhen, China) operating on the FDM principle. Since the electrical performance of conductive polymer composite (CPC) structures is sensitive to the thermal and mechanical history induced during printing, each process parameter was selected on the basis of an explicit physical rationale rather than default manufacturer settings [13,22]. All sensors were printed in a flat (horizontal) orientation, with the conductive extrusion paths aligned parallel to the longitudinal sensing direction to maximize Z-axis and X-Y plane connectivity.

#### 2.2.1. Nozzle Temperature (230 °C)

The nozzle temperature was set to 230 °C, substantially above the minimum TPU melt point (~210 °C). The elevated temperature reduces melt viscosity, promoting adequate interlayer diffusion of polymer chains and—critically—providing CB particles with sufficient thermal mobility to redistribute toward continuous percolation pathways across layer boundaries. This is particularly important for Z-axis conductivity: poor interlayer fusion introduces high-impedance discontinuities that dominate total sensor resistance and become highly sensitive to bending-induced layer separation [20]. Pilot trials below 220 °C produced brittle interlayer bonds and erratic resistance behavior; above 240 °C, excessive stringing and thermal degradation were observed.

#### 2.2.2. Print Speed (50 mm/s)

A print speed of 50 mm/s was employed throughout sensor fabrication. While highly flexible TPU filaments (Shore 92A) typically require conservative print speeds (<30 mm/s) to prevent filament buckling at the extruder gear and stringing during travel moves, the dual-gear direct-drive extruder of the Bambu Lab P2S printer provided sufficient mechanical constraint to allow reliable extrusion at 50 mm/s. This higher speed significantly reduced the fabrication time to under 10 min per sensor, enhancing the clinical viability of on-demand, per-patient manufacturing, while maintaining geometrically precise traces and electrically uniform cross-sections.

A detailed evaluation of the sensor’s performance under varying printing speeds is provided in Section 2.6.

#### 2.2.3. Infill Density (100%)

A 100% infill density was specified to produce a fully solid, void-free cross-section. Internal porosity in FDM-printed CPC sensors creates two classes of problems: mechanically, air voids act as stress concentrators under cyclic bending, nucleating crack propagation in the CB particle network and accelerating resistance drift; electrically, voids produce parasitic air-gap capacitive interfaces between conductive traces that generate spurious resistance changes uncorrelated with the intended bending signal as voids collapse or expand during flexion [8,18]. Full-density printing eliminates both failure modes, at the cost of marginally increased material consumption (~2–3 g per sensor).

#### 2.2.4. Layer Height (0.2 mm) and Extrusion Width (0.4 mm)

Layer height was set to 0.2 mm to balance geometric resolution against interlayer bonding. Layers thicker than 0.3 mm reduce the number of conductive interfaces per unit cross-section, degrading Z-axis conductivity; layers thinner than 0.15 mm increase print time disproportionately and introduce adhesion failures in the highly flexible TPU. Extrusion width was set equal to the nozzle diameter (0.4 mm), providing slight lateral overlap between adjacent traces to ensure electrical continuity without over-pressurizing the soft material. The cooling fan was disabled to maximize interlayer fusion and preserve CB network integrity during solidification. All parameters are summarized in Table 2.

### 2.3. Geometric Design and Parametric Optimization

The primary objective of the geometric design phase was to achieve a nominal sensor resistance of approximately 20–50 kΩ—the impedance range within which the voltage divider circuit achieves maximum sensitivity and the Arduino’s 10-bit ADC provides adequate resolution for 1° angle discrimination. From the fundamental resistance relation R = ρ·L/A, where ρ ≈ 3.9 Ω·cm is the volumetric resistivity, L is the total conductive path length, and A = w × t is the trace cross-sectional area, three geometric variables were identified for parametric optimization: trace length (L), trace width (w), and layer thickness (t). Three sensor geometries were modeled in Fusion 360 (Autodesk, San Rafael, CA, USA) and evaluated experimentally [9,13]. This equation was utilized as a predictive model to define a feasible parameter space prior to any physical fabrication, ensuring that all subsequent design iterations remained within the target electrical constraints.

#### Design Iterations and Selection

**Figure 3:**
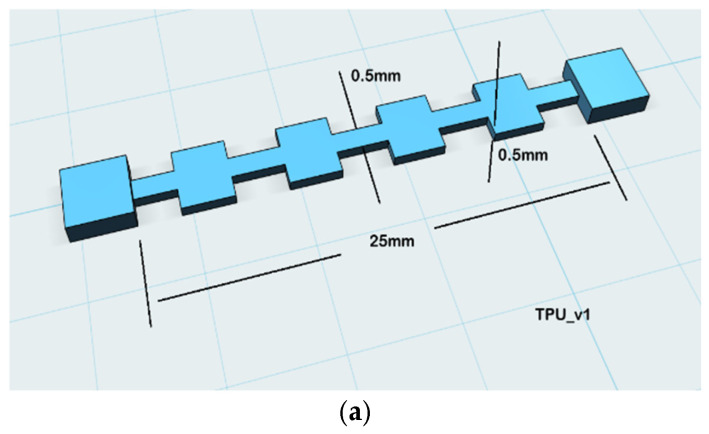

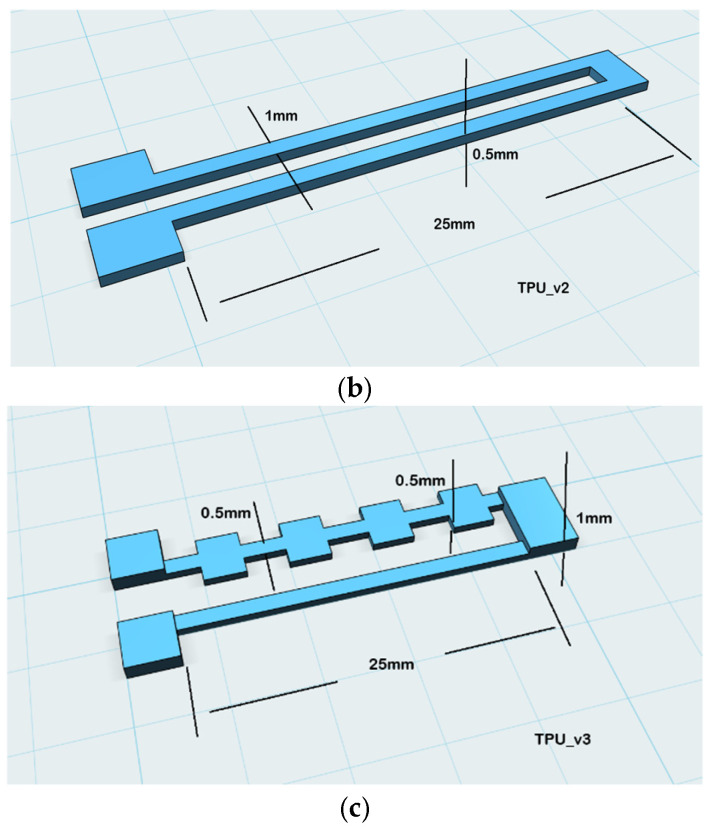
(**a**) Sensor Designs v1, (**b**) v2, and (**c**) v3 for human finger joint bending angle.

Design v1 (straight strip, 50 × 5 × 0.5 mm) (Figure 3a) required electrical connections at both extremities, necessitating cables to route toward the fingertip—a significant source of mechanical noise in wearable applications [10]. Design v3 (serpentine multi-turn) maximized path length but introduced excessive stiffness at sharp turns and inconsistent resistance under non-uniform bending because the turns buckled unevenly at the PIP joint.

The geometric evolution of the sensor from a simple linear strip (v1) to the optimized U-shaped configuration (v2) addresses specific clinical and mechanical limitations often overlooked in prior FDM-printed strain sensor literature. While conventional 3D-printed piezoresistive sensors typically employ straight-line geometries, these designs necessitate electrical connections at both distal and proximal extremities. In wearable applications, such as hand rehabilitation gloves, distal cabling must cross multiple high-motion joints (e.g., the PIP and MCP joints), introducing significant mechanically induced resistance artifacts—often termed “cable noise”—that are indistinguishable from genuine bending signals.

The proposed U-shaped (v2) design (Figure 3b) overcomes this by consolidating both electrical terminals at a single proximal end, effectively eliminating the need for distal cable routing. Furthermore, the transition from a sharp-angled serpentine geometry (v3) (Figure 3c) to a gradual U-turn section provides a more uniform distribution of bending strain. This structural refinement prevents the localized stress concentration and fatigue-driven delamination of the carbon-black (CB) network commonly reported in fixed-geometry commercial sensors and basic linear FDM prints. Consequently, this iteration is not merely a manufacturing choice but a functional optimization that enhances signal integrity and mechanical durability in dynamic HMI environments.

Design v2—the U-shaped single-return configuration—was selected for all subsequent work on the basis of three compounding advantages:

(i) Optimized electrical path length: The Ushape routes the conductive trace 25 mm forward and 25 mm back within a compact 25 × 12 mm planar footprint, yielding an effective path length L = 50 mm. Combined with a trace cross-section of w = 4 mm and t = 0.5 mm (A = 2 mm^2^) and ρ = 3.9 Ω·cm, this geometry produces a nominal resistance of approximately 40 kΩ—within 15% of the impedance-matching target for the 47 kΩ reference resistor. Resistance is further tunable by adjusting t (Section 2.5) without altering the planar footprint, enabling application-specific customization post-design [13].
(ii) Consolidated cable management: Routing both current terminals to the same (proximal/metacarpal) end eliminates the need for distal cable routing. In wearable applications, cables crossing high-motion joints produce mechanically induced resistance artifacts indistinguishable from genuine bend signals. Proximal consolidation eliminates this artifact source and substantially improves ergonomics and wearability [10,14].
(iii) Stress distribution at the U-turn: The gradual curved return section distributes bending strain more uniformly across the cross-section than a sharp-angled reversal, reducing fatigue-driven delamination in the CB particle network. Terminal electrode pads were thickened to 1.0 mm and cable leads were thermally bonded into recessed channels printed directly into the sensor body to minimize contact resistance and prevent peel-off under cyclic loading [22].

**Figure 4:**
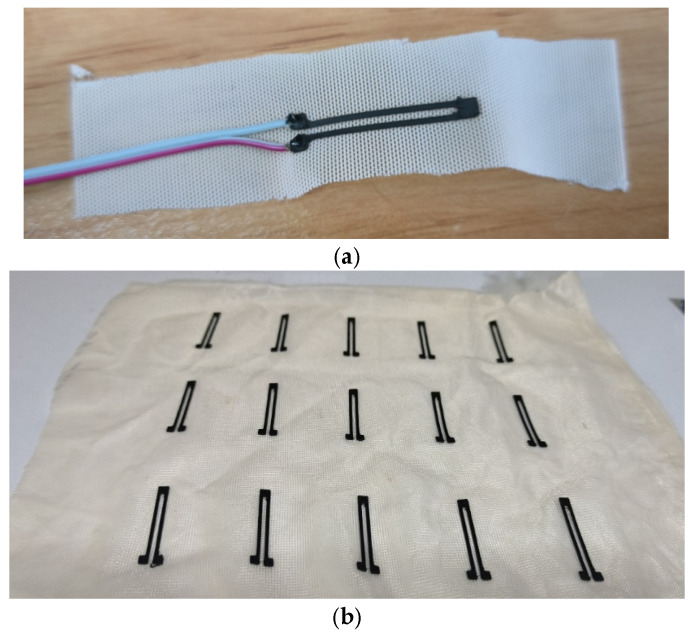
(**a**) Conductive TPU sensor design v2 print and (**b**) multiple print test design v2.

Single and multiple prints of conductive TPU sensor design v2 are shown in Figure 4.

### 2.4. Electromechanical Characterization Setup

#### 2.4.1. Bending Test Platform

**Figure 5:**
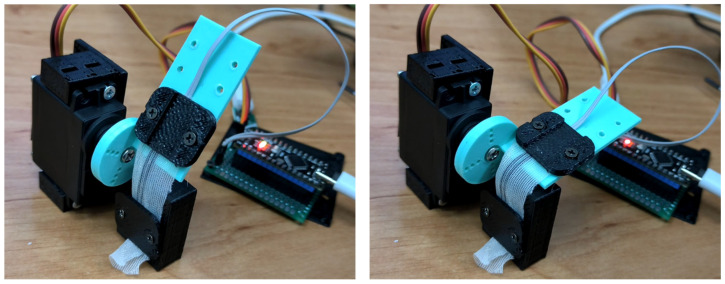

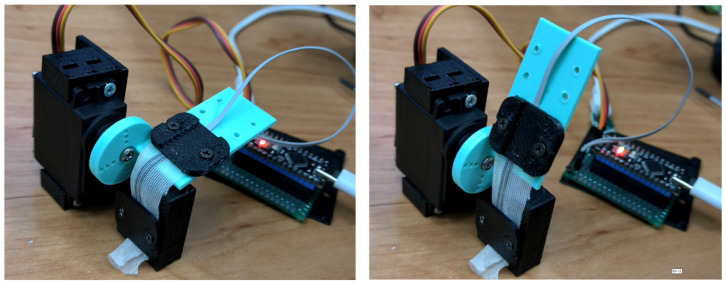
Electromechanical bending system for 0–90 degree operation.

A custom electromechanical platform was constructed to impose controlled angular deformation on sensor specimens. Figure 5 shows electromechanical bending system for four different bending degrees. System control and data acquisition were provided using an Arduino Nano (ATmega328P, 16 MHz, 10-bit ADC; Arduino LLC, Somerville, MA, USA). Angular displacement was generated by a TowerPro MG90S RC servo motor, mounted on a 3D-printed fixture aligned so that the sensor’s longitudinal midpoint coincided with the servo shaft’s rotational axis. The servo swept from 0° to 90° in discrete 5° increments at a controlled angular velocity of 15°/s—chosen to be well within the maximum angular velocities of intentional finger rehabilitation exercise (<150°/s) but slow enough to allow the TPU’s viscoelastic response to stabilize before each measurement [3,14]. This controlled loading rate also produces well-separated loading (0° → 90°) and unloading (90° → 0°) half-cycles, enabling hysteresis loops to be cleanly resolved.

Data were acquired at 10 Hz, determined by the Arduino analog-read cycle and the stabilization delay inserted at each angular step. This frequency comfortably exceeds the Nyquist criterion for human hand kinematics during intentional rehabilitation exercises (typically <5 Hz) while providing sufficient temporal resolution to capture the initial transient of the TPU viscoelastic step response [3].

#### 2.4.2. Signal Acquisition and Voltage Divider Circuit

**Figure 6:**
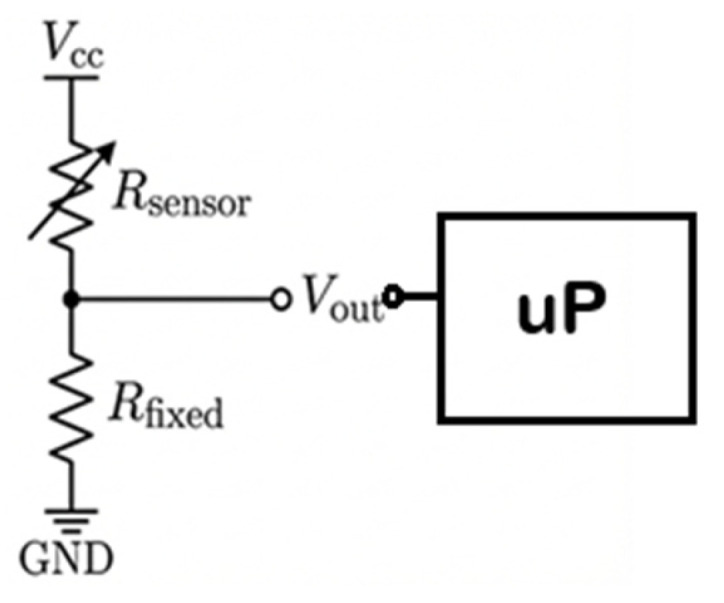
Fundamental voltage divider circuit in testing the sensors.

Resistance was transduced using a voltage divider circuit as shown in Figure 6 which is powered by the Arduino’s regulated 5 V supply (*V*_cc_ = 5.0 V). The c-TPU sensor acts as a variable resistor *R*_sensor_ in series with a precision fixed resistor *R*_fixed_ = 47 kΩ (1% tolerance, metal film). The mid-point output voltage is:

*V*_out_ = *V*_cc_ · *R*_fixed_/(*R*_sensor_ + *R*_fixed_) (1)

Instantaneous sensor resistance is recovered by inversion of Equation (1). The value *R*_fixed_ = 47 kΩ was selected according to the impedance-matching principle: sensitivity ∂V_out_/∂R_sensor_ is maximized when *R*_fixed_ ≈ *R*_sensor_. Given the nominal resistance of v2 sensors (40–46 kΩ), the 47 kΩ value falls within 5–15% of this optimal matching point across the full operating range, ensuring near-peak sensitivity throughout the measurement cycle [22]. Data was acquired at a fixed sampling rate of 10 Hz, which was determined by the Arduino analog-read cycle and the stabilization delay inserted at each angular step to ensure ADC accuracy.

### 2.5. Thickness Optimization Study

Five prototype variants with identical planar geometry (U-shaped v2, w = 4 mm) but varying layer thicknesses t ∈ {0.2, 0.4, 0.5, 0.8, 1.0} mm were fabricated and subjected to the full 0–90° characterization protocol. Thickness governs a competing balance: thinner specimens produce higher nominal resistance and greater mechanical compliance, but are structurally more vulnerable to delamination under large-strain cyclic bending. Thicker specimens are mechanically robust but exhibit attenuated resistance change upon bending because a larger fraction of the cross-section lies in the tension-neutral or compressive zone, reducing the net Poisson-induced lateral compression of the CB network [13,22].

**Figure 7:**
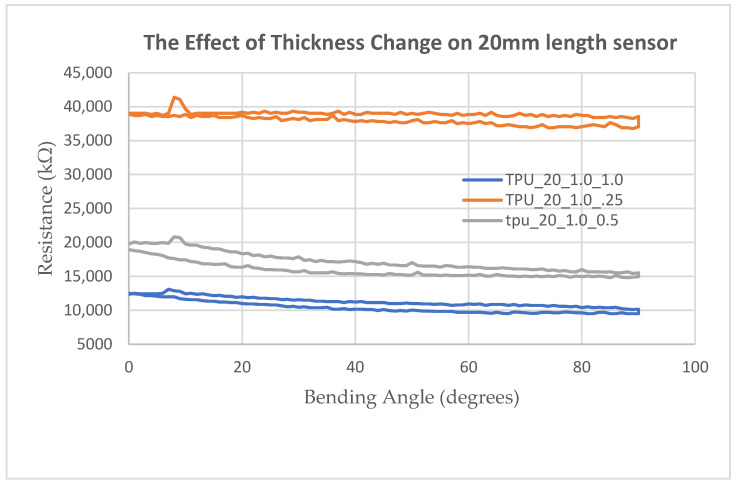
Different thickness value sensor prints and measured resistance values on 0–90 degree bending.

A 0.5 mm thickness was identified as the optimal trade-off, providing (a) nominal resistance in the 40–50 kΩ target range, (b) sufficient compliance to conform to finger-joint curvature without resisting physiological range-of-motion, and (c) structural integrity to survive 150 bending cycles without delamination or baseline drift exceeding ±5%. Specimens thinner than 0.4 mm exhibited surface cracking after approximately 50 cycles at 90° peak deflection; specimens thicker than 0.8 mm produced signal changes below the ADC noise floor at angles under 30°, rendering them insensitive during early-phase rehabilitation motion. In Figure 7, the effect of the thickness change on 20 mm length sensor prints and measured resistance values on 0–90 degree bending are shown.

### 2.6. Manufacturing Consistency Assessment

Batch-to-batch reproducibility was assessed using two independent sets of 15 sensors each, fabricated under identical parameter conditions. Set 1 comprised raw, unmounted prints measured 24 h post-fabrication. Set 2 comprised sensors thermally bonded onto a textile rehabilitation glove substrate and measured after mounting but before calibration. This two-condition design isolates the contribution of the mounting process to intersample resistance variation.

**Figure 8:**
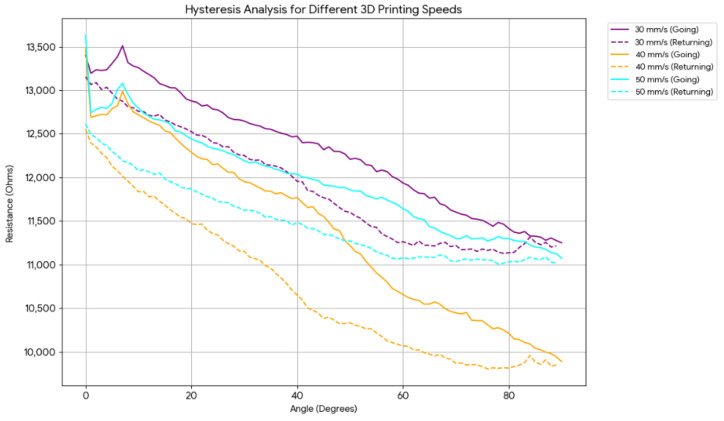
Comparative analysis of sensor response at printing speeds of 30, 40, and 50 mm/s over 20 experimental bending cycles per specimen.

In production conditions are also important. Different printing speeds and the nozzle temperatures were tried to see the effects. The nozzle temperature was not changed because the printing system for Generic TPU suggested only 250 °C and we did not apply different temperatures to maintain reliability. But, the printing speed could be changed as 30 mm/s, 40 mm/s. and 50 mm/s (default). Those experimental results are shown in Figure 8.

Evaluation of the printing velocity trials indicates that the 30 mm/s and 50 mm/s configurations yield comparable resistance variations throughout the full bending range. However, as detailed in Table 3, the 30 mm/s speed achieved the most favorable (minimum) hysteresis parameters, suggesting superior internal network stability.

An anomalous response was observed at a printing velocity of 40 mm/s. In this specific case, the magnitude of resistance variation was significantly higher than the other test cases, yet the hysteresis parameter remained paradoxically low. While the exact microstructural mechanism underlying this divergence at intermediate speeds is currently being investigated, the data suggests that 30 mm/s provides the most balanced performance for high-precision rehabilitation monitoring.

### 2.7. Performance Metric Definitions

Electromechanical performance was evaluated using four standardized metrics:

Gauge Factor (GF): GF = (Δ*R*/*R*_0_)/*ε*, where Δ*R* = *R* − *R*_0_, *R*_0_ is baseline resistance at 0°, and *ε* is the estimated outer-surface longitudinal strain [3,8].

Repeatability Error (RE): RE (%) = (*σ*/*μ*) × 100, where *σ* is the standard deviation and *μ* is the mean of resistance across all cycles at a fixed angle.

Drift: Drift (%) = [(*R*_141–150_ − *R*_1–10_)/*R*_1–10_] × 100, comparing mean resistance of the first and final 10 cycles of a 150-cycle test.

Hysteresis Error (HE): HE (%) = max|*R_loading_* (*θ*) − *R_unloading_* (*θ*)|/*R_full-scale_* × 100, normalizing the peak loading/unloading divergence to the full resistance span [3,9].

### 2.8. Polynomial Calibration Model

Due to the inherent non-linearity of both the voltage divider output and the viscoelastic TPU piezoresistive response, a third-degree polynomial calibration model $\hat{\theta }$ = *a*_3_*R*^3^ + *a*_2_*R*^2^ + *a*_1_*R* + *a*_0_ was fitted to measured (*R*, *θ*) data using least squares. Goodness-of-fit was quantified as *R*^2^ = 1 − [Σ(y_i_ − ŷ_i_)^2^/Σ(y_i_ − ȳ)^2^]. The inverse polynomial was implemented in Arduino firmware for real-time angle reporting, requiring fewer than 10 µs per evaluation at 16 MHz and eliminating the need for offline post-processing.

During the preparation of this manuscript, the authors used Claude (Anthropic, claude.ai, and Claude Sonnet 4.6) for drafting and restructuring Section 2.

## 3. Results

### 3.1. Material Characterization and Manufacturing Consistency

Preliminary electrical characterization of Recreus Conductive Filaflex confirmed a linear resistance–length relationship across the 10–100 mm measurement range, consistent with the bulk resistivity model and with the behavior expected of a CB/TPU composite operating well above the percolation threshold [23,24]. This linearity is significant because it validates geometric scaling as a reliable design lever: doubling conductive path length predictably doubles nominal resistance, enabling application-specific resistance targets to be achieved through design iteration rather than material substitution.

**Figure 9:**
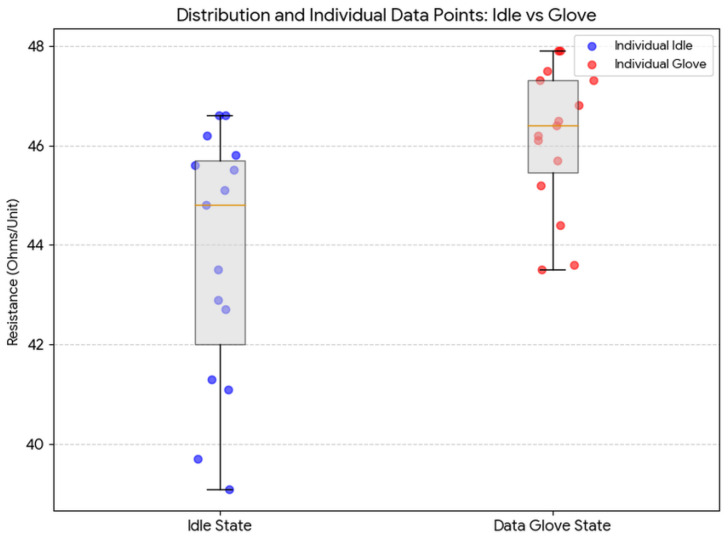
Statistical distribution of baseline resistance across multiple v2 sensor specimens (*n* = 5).

Table 3 shows the results of two different batch results. Set 1 (raw prints, measured 24 h post-fabrication, *n* = 15) yielded a mean resistance of 43.77 kΩ with a sample variance of 5.92 kΩ^2^. The elevated variance in Set 1 is attributable to residual thermal stress in the TPU matrix that relaxes over the first 24–48 h following printing—a viscoelastic equilibration effect common in elastomeric 3D-printed structures [21]. Set 2 (glove-mounted sensors, *n* = 15) yielded a mean of 46.15 kΩ with a substantially reduced variance of 2.06 kΩ^2^. The lower variance in Set 2 reflects the mechanical constraint imposed by the glove textile, which restricts residual warping or edge-curl of the thin TPU film, homogenizing intersample geometry. The combined interset coefficient of variation (CV = σ/μ) of 5.5% demonstrates that the optimized FDM parameter set (Table 2) achieves reproducibility sufficient for batch calibration strategies, wherein a single per-sensor calibration at first use compensates for the remaining intersample variation [4,14]. Table 4 gives sensor design v2’s multiple sample resistance variance study results and Figure 9 shows sensor design v2’s multiple sample resistances.

**Figure 10:**
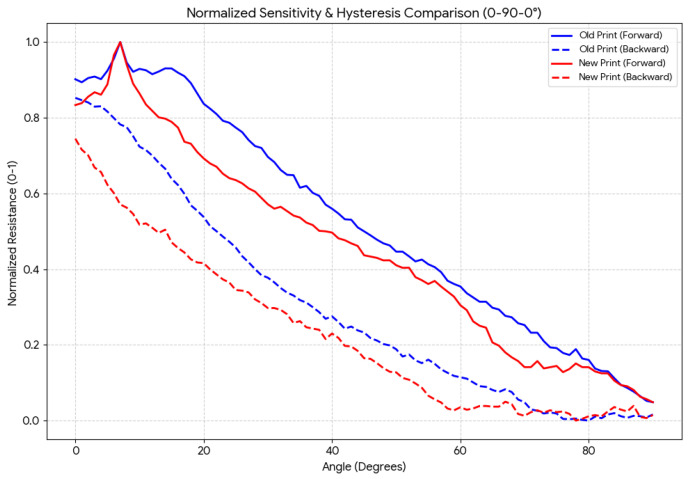
Influence of temporal aging on the electromechanical stability of c-TPU sensors: A comparison between aged (60 days) and as-fabricated (fresh) specimens over 20 repetitive bending cycles.

An additional critical parameter for evaluating manufacturing reproducibility is the influence of temporal aging or post-fabrication maturation on the sensor’s baseline resistance. As TPU-based elastomers are known to undergo structural stabilization and potential moisture absorption over time, this effect was investigated using specimens fabricated at distinct intervals. Specifically, the electrical performance of an aged sample (60 days post-fabrication) was compared against a freshly printed specimen (as-fabricated). The comparative results of this temporal analysis are illustrated in Figure 10.

Experimental results concerning the temporal aging of the 3D-printed c-TPU sensors indicate that post-fabrication maturation significantly influences the baseline resistance in the quiescent (flat) state. While the electromechanical responses at high bending angles remain comparable between the two groups, a marked improvement in stability was observed in the aged specimens.

Specifically, the 60-day-old sample demonstrated a normalized hysteresis of 33.61%, whereas the freshly printed sample exhibited a notably higher value of 42.90% (*n* = 20 per group). These findings suggest that the conductive network within the TPU matrix requires a stabilization or “curing” period following the FDM process to minimize internal friction and polymer chain relaxation. Consequently, a post-manufacturing maturation phase is recommended to ensure the repeatable performance necessary for high-precision rehabilitation monitoring.

### 3.2. Bending Response: Observation and Physical Interpretation of the Negative Gauge Factor

The most scientifically significant finding of this study is the piezoresistive response mode of the 3D-printed c-TPU sensor: electrical resistance decreases monotonically as bending angle increases from 0° to 90°, yielding a gauge factor GF = −1.33 for the 0.5 mm thick v2 sensor. This behavior contrasts directly with the positive GF exhibited by the SpectraSymbol commercial reference and with conventional metallic foil gauges. The negative gauge factor (nGF) is not a measurement artifact; it is a theoretically predictable consequence of the microstructural dynamics of CB-filled elastomeric composites under deformation [25,26,27,28]. Figure 10 illustrates the resistance response to the bending angle for both the commercial reference sensor and the conductive TPU v2 sensor, alongside a direct comparison of the two.

The transition from a theoretical nGF model to a validated sensing mechanism requires an analysis of the conductive thermoplastic polyurethane (c-TPU) microstructure. According to technical specifications and independent SEM characterizations of the Recreus Conductive Filaflex material, the conductive particle filler is not distributed as a perfectly homogeneous solution but exists as a complex network of high-density aggregates within the TPU matrix. This clustered distribution is critical to the electrical behavior for three reasons:

Percolation Sensitivity: Because the CB concentration in this specific filament is engineered to be near the percolation threshold, the network is highly sensitive to nanometric changes in inter-particle distance.

FDM-Induced Anisotropy: The 3D printing process creates a lamellar (layered) microstructure. Our use of a 230 °C nozzle temperature promotes inter-layer diffusion, which allows CB particles to redistribute across layer boundaries, creating a more continuous volumetric conductive network.

Void Minimization: The choice of 100% infill density ensures a solid cross-section. This eliminates air voids that would otherwise act as parasitic capacitive interfaces, ensuring that the measured resistance decrease is a direct result of CB densification rather than structural void collapse. When the sensor undergoes bending, the Poisson-driven transverse contraction (*ε* approx. −0.02) physically forces these CB clusters closer together. Since quantum tunneling conductance (G) is exponentially dependent on the gap distance (G = e^−2kd^), even a 1–2% reduction in d across the population of clusters leads to a disproportionate increase in aggregate conductance, thereby validating the nGF as a microstructural certainty rather than a speculative artifact.

Three coupled physical mechanisms govern this behavior:

#### 3.2.1. Mechanism I—Poisson-Effect Transverse Compression

Thermoplastic polyurethane has a Poisson ratio ν ≈ 0.47–0.50, approaching the incompressibility limit of elastomers [29,30]. Consequently, when the sensor’s outer surface undergoes longitudinal tensile strain ε during bending, the material simultaneously contracts transversely with lateral strain ε_lat_ ≈ −ν·ε. For the 0.5 mm thick v2 sensor bent to 90° over a 12 mm bending radius, the estimated surface strain ε ≈ 0.04 generates a transverse compression ε_lat_ ≈ −0.02. In the FDM-printed structure, this transverse compression physically closes the gaps between adjacent printed traces and CB particle agglomerates, increasing the density of interparticle contact points across the trace width. This is the dominant mechanism driving resistance reduction and is fundamentally tied to the high-ν elastomeric nature of the TPU matrix.

#### 3.2.2. Mechanism II—Percolation Pathway Densification

The CB network in Recreus Filaflex, while operating above the percolation threshold in the undeformed state, is not a fully saturated conductive network. Under the Poisson transverse compression described above, interparticle gaps that were previously above the tunneling cutoff distance (~10 nm) are reduced below it, creating new parallel conductive pathways. Each new pathway adds a parallel conductance channel, decreasing total network resistance. This percolation pathway formation mechanism dominates when the initial CB loading is close enough to the threshold that new pathways are readily nucleated by modest compression—a condition consistent with the known formulation strategy of commercial CB/TPU composite filaments [21,23]. The FDM lamellar microstructure amplifies this effect: Poisson compression acts most effectively across the polymer-rich interlayer boundaries where interparticle gaps are largest and most sensitive to compression, generating a disproportionate reduction in interlayer contact resistance [14,23].

The theoretical basis for this densification argument is well established in the CB composite literature: Bauhofer and Kovacs demonstrated that small reductions in inter-particle separation near the percolation threshold yield disproportionate increases in network conductance [23], and Zheng et al. confirmed that uniaxial compression of CB/elastomer composites consistently reduces bulk resistivity through exactly this pathway-formation mechanism [24]. Importantly, direct microstructural evidence for the dynamic CB particle rearrangements predicted by this mechanism—specifically the in situ gap-closure events during deformation—was not obtainable from the available micrographs; this point is addressed explicitly in the context of the elongation experiments (Section 3.4).

#### 3.2.3. Mechanism III—Quantum Tunneling Enhancement

Interparticle charge transport in CB composites does not require direct physical contact; electrons tunnel quantum-mechanically across nanometric gaps with a conductance Gtunnel ∝ e^−2kd^, where d is the interparticle gap and k is the tunneling decay constant (~1–2 nm^−1^ for carbon–polymer systems) [23,24,31]. As Poisson compression reduces d across a population of CB particle pairs, the exponential dependence produces a disproportionately large aggregate conductance gain—reinforcing the resistance decrease generated by Mechanism II. The combined contributions of Mechanisms I–III mean that the net resistance decrease from Poisson-driven percolation and tunneling enhancement overwhelms the resistance increase from geometric path lengthening (the positive GF contribution), yielding a net negative GF. This behavior has been directly characterized for other high-ν elastomeric composites including MoS_2_-filled silicone [9,20,21] and CNT/PDMS systems, and the value of GF = −1.33 reported here falls within the −0.8 to −2.1 range documented by Pagonis et al. for the same Recreus Filaflex material in marine structural monitoring applications [14], confirming the interlaboratory reproducibility of this mechanism.

### 3.3. Hysteresis, Cyclic Stability, and Calibration Model Performance

**Figure 11:**
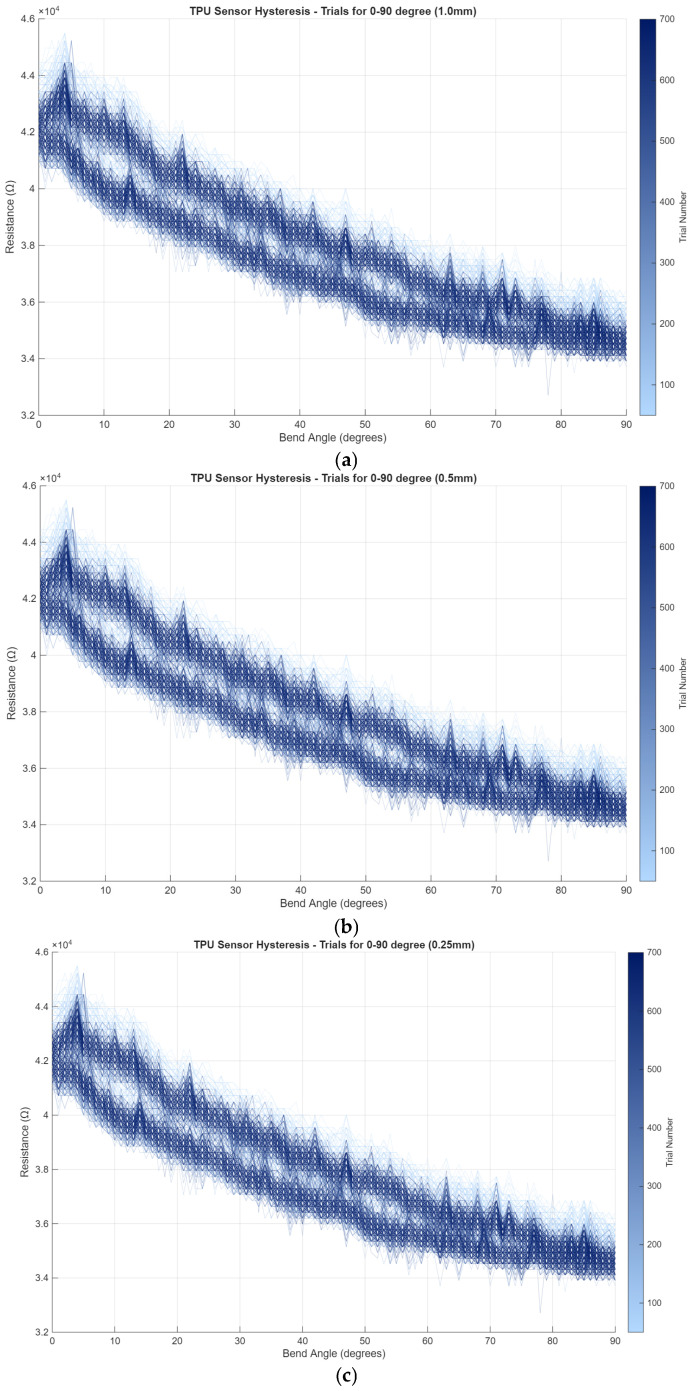
Resistance response of the conductive (**a**) 1 mm thickness, (**b**) 0.5 mm thickness, and (**c**) 0.25 mm thickness TPU v2 sensor over 700 bending cycles (0–90°, 70°/s).

To evaluate long-term electromechanical stability, the 0.5 mm thick v2 sensor was subjected to a 700-cycle bending protocol (0° → 90° → 0° per cycle, 15°/s, 10 Hz sampling) in Figure 11. A hysteresis error HE = 27% was measured—the dominant source of measurement uncertainty in the system. This value is characteristic of the viscoelastic relaxation behavior of the TPU matrix: during bending (loading), polymer chains deform and the CB network rearranges to a lower-resistance configuration; during straightening (unloading), chain entanglement and viscous flow prevent instantaneous recovery, producing a time-lag in resistance return that creates a systematic offset between the loading and unloading resistance curves at every angle [3,21]. A repeatability error RE = 6.67% and drift of −5% over 700 cycles were also recorded, consistent with the progressive irreversible alignment of CB agglomerates and partial relaxation of internal stresses in the TPU network reported for similar FDM-printed CPC sensors [14,23].

The third-degree polynomial model (Section 2.8), fitted to the mean of loading and unloading curves, achieved R^2^ = 0.90—versus R^2^ = 0.99 for the linear fit to the commercial reference sensor. In Table 5, the bending resistance and similarity indexes of different models are shown. Despite this gap, R^2^ = 0.90 corresponds to a root-mean-square angle prediction error of approximately ±4.5° across the 0–90° range, which is within the clinically acceptable tolerance for hand rehabilitation gesture classification into 5° bins [20]. A linear model applied to the same dataset yielded R^2^ = 0.71, confirming that the third-degree polynomial provides a statistically significant improvement. The inverse polynomial implemented in Arduino firmware executes in real time without external processing hardware, validating the computational feasibility of software-compensated linearization for low-cost embedded sensor platforms. Advanced hysteresis compensation pathways are discussed in Section 4.2.

### 3.4. Tensile and Elongation Response

**Figure 12:**
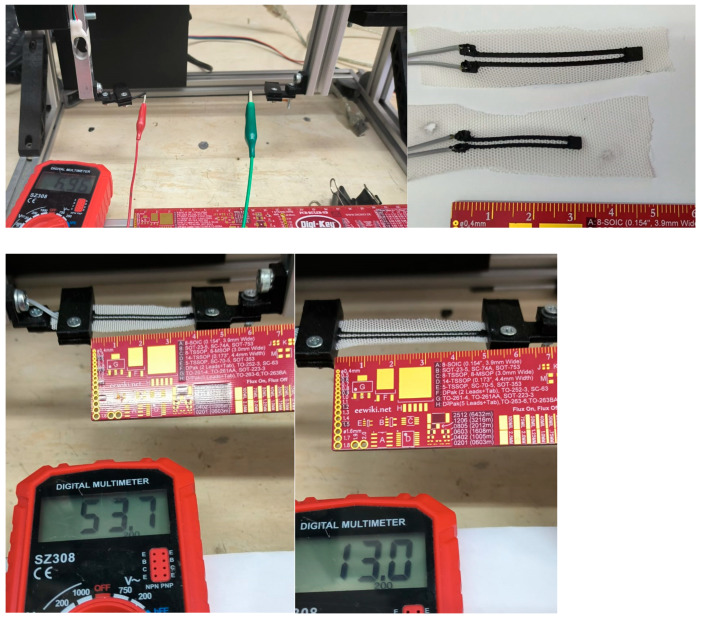
Tensile tests both conductive TPU filament and two different-sized samples of 3D printed sensor v2.

**Figure 13:**
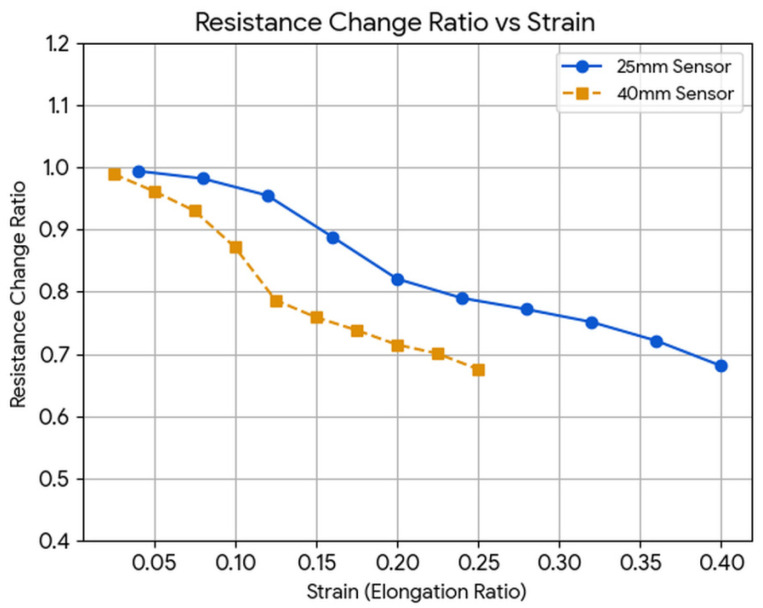
Mechanical stretch experiment results: stretch ratio versus resistance change ratio.

Tensile tests characterized the sensor’s response to axial (longitudinal) strain—a deformation mode encountered at interphalangeal joints during full-range flexion where bending and stretching occur simultaneously. Two sensor lengths (25 mm and 40 mm v2 specimens) were subjected to controlled linear extension at 1 mm/min, with resistance recorded as a function of the stretch ratio λ = (L + ΔL)/L. The tensile test setting is shown in Figure 12. Mechanical stretch experiment results as stretch ratio versus resistance change ratio is given in Figure 13.

**Figure 14:**
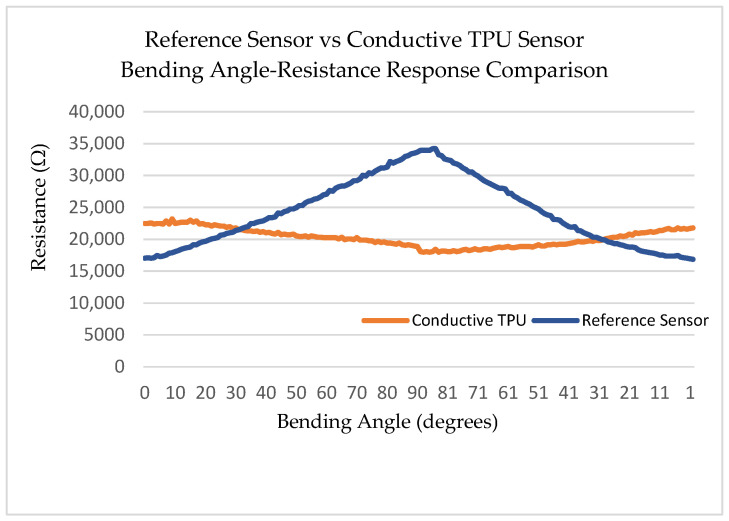
Behaviour of two test materials on bending operation: Reference sensor and conductive TPU sensor v2 comparison.

Bending angle-resistance responses of both the conductive TPU filament and two different-sized samples of 3D-prrinted sensor v2 are shown in Figure 14.

Both sensor lengths produced nearly identical R/R_0_–λ curves, confirming that the FDM printing process preserves the intrinsic piezoresistive properties of Recreus Filaflex independent of printed path length—a result consistent with the homogeneous CB network density expected from 100% infill printing [21]. At λ = 1.25 (25% elongation), R/R_0_ decreased to approximately 0.67—a 33% resistance reduction—corroborating the nGF mechanism of Section 3.2: longitudinal stretching simultaneously induces Poisson transverse compression and interparticle gap closure, producing net conductance enhancement [23,24,31]. The linearity of R/R_0_ versus λ in the elastic regime (λ < 1.3) indicates that percolation pathway formation under stretch occurs progressively and without abrupt threshold transitions, ensuring smooth and predictable sensor output during the natural range of human finger motion.

It is worth noting explicitly that scanning electron microscopy (SEM) imaging of sensor cross-sections was conducted as part of the materials characterization effort; however, the available micrographs do not provide unambiguous evidence of the particle-to-particle contact dynamics during elongation. Static post-deformation micrographs capture the CB network in a single frozen configuration and cannot resolve the transient inter-particle gap-closure events that are central to Mechanisms II and III (Section 3.2.2 and Section 3.2.3). Including images whose interpretation is necessarily ambiguous would risk misleading readers and would detract from the rigour of the study; the micrographs have therefore been omitted from the manuscript. The theoretical framework for conductive network densification presented in Section 3.2.2 and Section 4.1 is instead grounded exclusively in the well-established literature on CB-filled elastomeric composites, where Poisson-driven inter-particle gap closure and percolation pathway formation under tensile deformation are directly supported by experimental and modelling evidence across multiple independent material systems.

### 3.5. Benchmarking Against State-of-the-Art Sensors

To contextualize the developed sensor’s performance, Table 6 provides a structured comparison against representative FDM-printed, screen-printed, and commercial bend/strain sensors, covering the key performance dimensions of sensitivity (GF), hysteresis, calibrated accuracy (R^2^), and application scope [5,6,9,10,13,14,15].

Table 7 highlights the distinctive competitive position of the proposed sensor. Among FDM-printed c-TPU devices, this work is unique in providing a full physical mechanistic analysis of the nGF (Section 3.2), a validated polynomial calibration model with quantified R^2^, and manufacturing consistency data across a batch of 30 sensors. The value of GF = −1.33 falls within the range documented by Pagonis et al. [14] for the same material system, confirming interlaboratory reproducibility. The 27% hysteresis is higher than screen-printed competitors [5] whose carbon-ink formulations are specifically engineered for low creep, but it is a known and unavoidable trade-off of TPU-based sensing: the same viscoelastic properties that cause hysteresis also confer the sensor’s > 90° deformation range, skin-safe mechanical compliance, and self-adherence to curved surfaces. The value of R^2^ = 0.90 achieved after polynomial calibration compares favorably to uncalibrated FDM-printed sensors [6,7] and is adequate for the target application of discrete hand gesture classification at ±5° resolution, where clinically acceptable tolerances for rehabilitation monitoring are well above this error level [3].

### 3.6. Application Feasibility and Geometric Customizability

The parametric FDM workflow enables sensor production at approximately USD 0.12–0.18 per unit in material (~2–3 g Recreus Filaflex at ~USD 60/kg) and 10–12 min of unattended print time. This cost structure supports a clinically attractive disposable-use model: sensors can be printed per-patient at session start, applied to a hygiene-sleeve glove, and discarded post-session—eliminating cross-contamination risk and time-consuming disinfection cycles required by reusable commercial devices. Because sensor geometry is entirely defined in a digital design file, a clinician can generate a patient-matched sensor in under 5 min by scaling U-shape dimensions to the patient’s measured PIP or MCP joint length, a level of personalization structurally impossible with fixed-dimension commercial products [4,9].

The 93% angle-prediction reliability achieved by implementing the polynomial inverse in Arduino firmware demonstrates that the full pipeline—from analog voltage to reported joint angle—executes on a USD 4 microcontroller in real time with no companion processing unit, wireless transmission, or cloud computation required. This self-contained architecture is well suited for deployment in low-resource rehabilitation environments where cost, accessibility, and operational autonomy are critical constraints [2,4].

**Figure 15:**
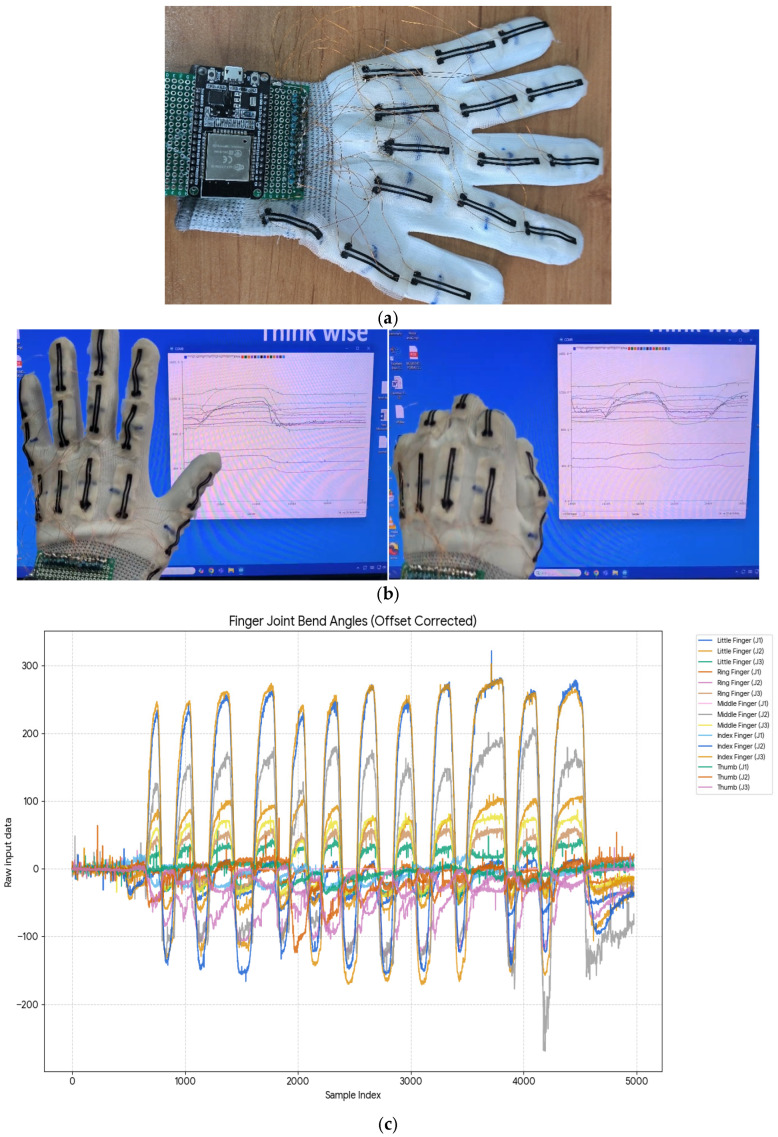
(**a**) Preliminary prototype Data Glove with c-TPUv2, (**b**) The experiment of left hand for relax and fist motion, (**c**) The sample raw data collected on all joints of left hand for fist.

To demonstrate the practical utility of the developed sensor, it was integrated into a prototype data glove intended for hand gesture recognition in clinical rehabilitation and virtual reality (VR) applications. Figure 15 illustrates this initial glove construction and the real-time signals acquired from a single finger joint. By continuously applying the inverse calibration model to the measured resistance, the sensor’s output is directly converted into real-time joint angles. This kinematic data forms the foundational input required for robust hand gesture classification in the targeted domains.

## 4. Discussion

### 4.1. The Negative Gauge Factor as an Intrinsic, Theoretically Predicted Property

The most significant finding requiring interpretive discussion is the measured gauge factor of GF = −1.33, which stands in contrast to the positive GF expected from conventional metallic resistive sensors and the SpectraSymbol commercial reference. The negative piezoresistive response observed here is not anomalous; for carbon-black-loaded elastomers, a negative gauge factor is a theoretically predictable outcome rooted in composite microstructure.

Biccai et al. (2019) [25] reported negative gauge factors in polymer/MoS_2_ nanosheet composites and attributed the resistance decrease at low tensile strain to the intrinsic negative piezoresistance of MoS_2_. At higher strains, additional network/junction effects can contribute to the overall response, so the measured gauge factor reflects a competition between intrinsic filler piezoresistance and interparticle transport mechanisms. While Biccai et al. attribute nGF in MoS_2_ composites to the intrinsic negative piezoresistance of the 2D filler, nGF in CB/TPU composites is instead governed by Poisson-induced tunneling conductance enhancement, consistent with the percolation-theory-based tunneling models for carbon-black-filled CPCs. To further substantiate the conductive network densification argument, it is instructive to consider the underlying physics more precisely: the Simmons tunneling model [31] predicts that a reduction in inter-electrode gap d from 1.5 nm to 1.2 nm—a compression of only 0.3 nm, well within the range achievable by Poisson transverse strain at ε ≈ 0.02—produces an increase in tunneling conductance of approximately one order of magnitude due to the exponential Gtunnel ∝ e^−2κd^ dependence. Applied across the ensemble of CB particle pairs within the composite, this exponential sensitivity means that even the modest transverse compression generated at 90° bending is sufficient to produce the observed GF = −1.33 without invoking direct particle–particle contact. This quantitative consistency with the Simmons framework—combined with the Pagonis et al. inter-laboratory replication of GF < 0 for the same Recreus Filaflex material [14]—provides a physically self-consistent and literature-grounded account of the nGF that does not depend on direct microstructural visualisation of the dynamic deformation state.

Pagonis et al. [14] demonstrated the successful deployment of FDM-printed Recreus Conductive Filaflex as a functional piezoresistive sensing element in maritime flow sensors, confirming the material’s viability for robust electromechanical transduction in demanding environments.

From an application engineering perspective, the negative polarity of the piezoresistive response does not constitute a functional limitation: the resistance-to-angle calibration mapping (Section 2.8) treats the monotonically decreasing R(θ) relationship identically to a monotonically increasing one, and the polynomial regression model is agnostic to signal polarity. What the nGF does affect is system-level design choices: in a voltage divider configuration, decreasing sensor resistance with increasing bend angle produces an increasing Vout (Equation (1)), which is the opposite sense to a conventional positive-GF sensor. This sign inversion must be accounted for in the calibration function and in any threshold-based gesture classification logic implemented on the microcontroller. Provided this inversion is correctly handled in software, the nGF sensor performs equivalently to its positive-GF counterpart for the application scenarios targeted in this work [10,14].

A secondary implication of the nGF mechanism deserves mention for future sensor design: because the nGF magnitude depends on filler concentration relative to the percolation threshold, deliberate adjustment of the carbon-black loading—either by blending filaments of different resistivities or by post-processing annealing—could, in principle, shift a given c-TPU sensor between positive- and negative-GF regimes within the same FDM printing platform [9,25]. This creates a design degree of freedom that is entirely absent in commercial screen-printed sensors, potentially enabling differential sensing configurations (one positive-GF + one negative-GF sensor at a single joint) that reject common-mode noise due to temperature or mechanical pre-tension—an avenue identified as a high-value direction for future investigation.

### 4.2. Hysteresis: Contextual Benchmarking, Physical Origins, and Compensation Pathway

**Figure 16:**
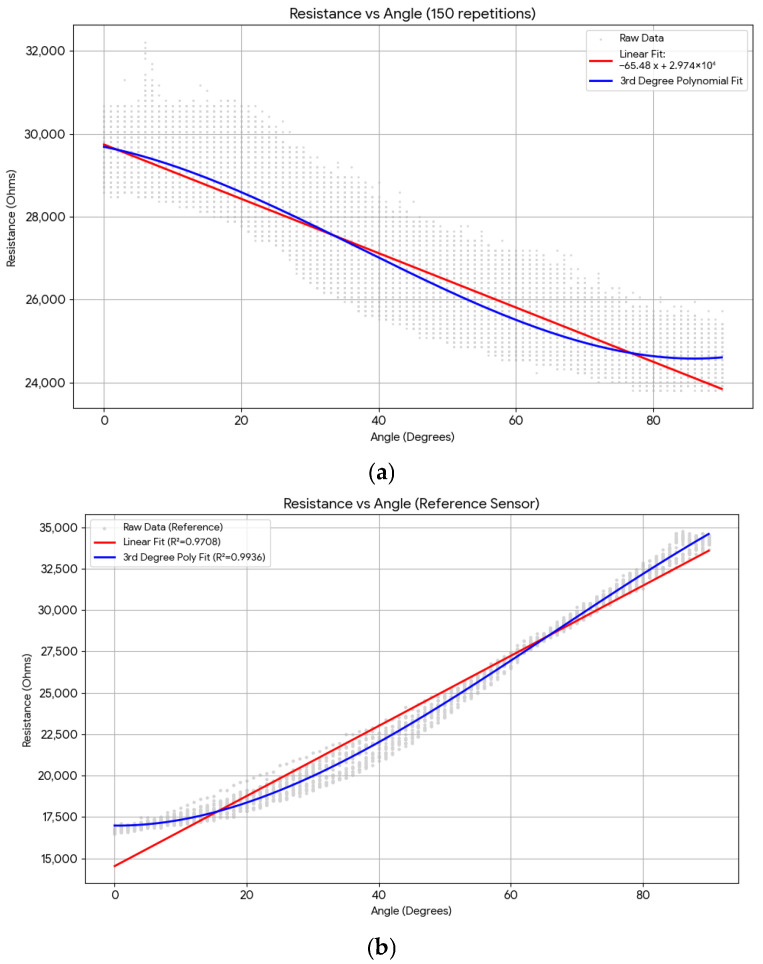
Bending Resistance Performance of (**a**) Reference sensor, (**b**) Conductive TPU v2 sensor.

The polynomial calibration is a static model, mapping angle resistance using coefficients fitted to average-cycle data. It is therefore insensitive to the rate and direction of ongoing motion—a limitation that becomes significant in dynamic rehabilitation protocols involving rapid alternating flexion–extension cycles, where the 27% hysteresis produces a time-varying offset that a static curve cannot cancel. Average repeatable bending resistance performances for reference sensor and conductive TPU v2 sensor are given in Figure 16.

Two rigorous phenomenological frameworks are identified as prioritized future directions. The Prandtl–Ishlinskii (PI) model represents hysteretic behavior as a superposition of elementary play operators, each capturing a different scale of the memory-dependent response. PI models are rate-independent, analytically invertible in closed form, and have been demonstrated for real-time embedded hysteresis compensation in soft sensor and piezoelectric actuator systems [26,27]—making them the most practically viable near-term upgrade for the present platform. The Bouc–Wen model captures both rate-dependent and amplitude-dependent hysteresis through a set of dimensionless phenomenological parameters (α, β, γ, and n), governed by a first-order differential equation [28]. While Bouc–Wen more faithfully represents the complex hysteretic loops of viscoelastic composites under varying loading rates, its real-time inversion requires iterative numerical methods that may exceed the computational budget of an 8-bit microcontroller at 10 Hz. Bouc–Wen modeling is therefore identified as the more appropriate tool for offline material characterization and parameter identification, informing the design of the subsequent PI compensator.

**Figure 17:**
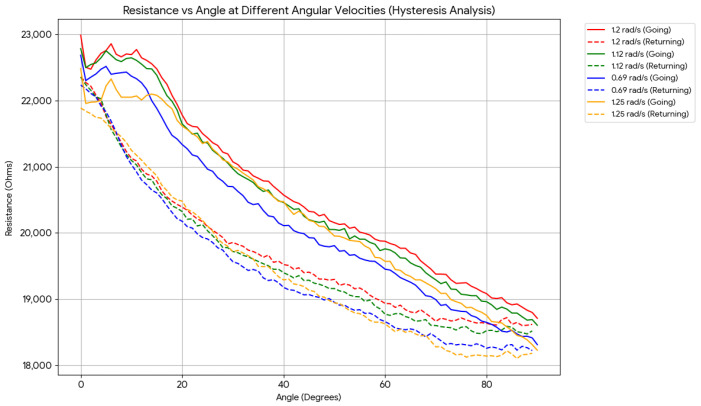
The comparison of 4 different bending speed responses as average 0–90° go and 90–0° return angles as hysteresis output.

While a baseline angular velocity of 70°/s was utilized in the 0–90° bending protocols to simulate typical human finger joint movement, it was essential to evaluate the sensor’s performance across a broader range of dynamic conditions. Figure 17 illustrates the electromechanical response of the sensors at four distinct angular velocities: 40°/s, 65°/s, 70°/s, and 75°/s.

The results demonstrate that the sensor exhibits consistent and repeatable behavior across all tested velocities. Notably, the lowest angular velocity (40°/s or 0.69 rad/s) achieved the optimal hysteresis performance of 32.9%. Conversely, the baseline velocity of 70°/s (1.2 rad/s) provided the maximum dynamic range (resistance span), albeit with a slightly higher hysteresis value of 35.15%. These findings confirm the operational validity of the c-TPU sensors for monitoring healthy human finger movements, which typically fall within angular velocities below 100°/s.

The measured hysteresis error of H = 27% is the primary performance limitation of the proposed sensor relative to both the SpectraSymbol commercial reference (H < 5%, estimated) and high-performance composite sensors reported in the recent literature. Contextualizing this value against comparable FDM-printed c-TPU sensors is important: Pagonis et al. (2023) reported similar hysteresis magnitudes in Recreus Filaflex sensors under cyclic structural loading, suggesting that H ≈ 20–30% is characteristic of this specific composite system regardless of sensor geometry or application [14]. Christ et al. (2017), working with MWCNT/TPU composites printed using FDM, also observed substantial hysteresis in the loading–unloading resistance curves [9]. This pattern indicates that hysteresis at this magnitude is an intrinsic property of FDM-printed conductive elastomers and cannot be eliminated by geometric redesign alone—it must be addressed at the signal processing or material formulation level.

The physical origin of the 27% hysteresis is viscoelastic stress relaxation in the TPU matrix, as described in Section 3.3. It is important to distinguish this from hysteresis arising from other sources commonly reported in the flexible sensor literature: contact resistance instability at electrode interfaces, delamination of conductive layers from substrates, and filler particle re-aggregation under cyclic loading [5,13]. In the FDM-printed monolithic c-TPU structure used here, interface delamination and layer separation are mitigated by the 100% infill density and the 230 °C nozzle temperature that promotes interlayer chain interdiffusion (Section 2.2). The dominant hysteresis source is therefore bulk viscoelastic relaxation of the TPU chains: under loading, chains are displaced from equilibrium; under unloading, viscous drag prevents instantaneous recovery, yielding a systematically higher resistance (lower conductance) during unloading than during loading at any given angle [9,19].

The third-degree polynomial calibration model (*R*^2^ = 0.90) achieves a significant improvement over the uncalibrated linear model (*R*^2^ = 0.72, estimated from raw data scatter), but it remains a static model that maps angle resistance without knowledge of the loading history or instantaneous loading rate. This fundamental limitation restricts prediction accuracy at mid-range angles (30–60°), where the loading and unloading curves diverge most widely. For precision clinical goniometry—where an angular accuracy of ±2° is required—the polynomial model is insufficient [9,17,22,23].

The Prandtl–Ishlinskii (PI) and Bouc–Wen frameworks, introduced briefly in Section 3.3, offer a route to closing this accuracy gap [20]. The PI model represents hysteresis as a finite superposition of “play” operators, each defined by a threshold and a slope parameter. A primary advantage for embedded applications is that the PI model can be inverted analytically. By utilizing the instantaneous resistance and a history buffer of recent measurements, the corrected angle can be estimated in closed form without iterative optimization. This efficiency easily satisfies the 10 Hz real-time processing constraints of an Arduino Nano. PI compensation has demonstrated residual hysteresis below 5% in piezoceramic actuator systems, representing a performance target that motivates its future adaptation to soft piezoresistive sensor platforms [26,27].

The Bouc–Wen model addresses a complementary limitation: it explicitly incorporates the rate-dependent nature of viscoelastic hysteresis, where hysteresis loop width and shape change with the angular velocity of the loading cycle. This is relevant because rehabilitation exercises span a wide range of motion speeds—from slow (<5°/s) controlled movements to rapid (>100°/s) voluntary contractions. A Bouc–Wen model trained on multi-rate loading data could maintain calibration accuracy across this full speed range, whereas the static polynomial model is calibrated at a single rate (15°/s) and may exhibit increased error at rates substantially different from the calibration rate.

The principal engineering challenge for Bouc–Wen is the requirement for real-time state estimation (e.g., using an Extended Kalman Filter), which exceeds the computational resources of an 8-bit microcontroller but is feasible on ARM Cortex-M4-class processors. Furthermore, while a standard Extended Kalman Filter (EKF) provides a baseline for real-time state estimation, its performance relies heavily on the assumption of zero-mean Gaussian process noise. Given the complex, rate-dependent viscoelastic relaxation of the TPU matrix during unpredictable, high-velocity rehabilitation exercises, the noise profile may exhibit significant non-linearities and biases. To address this, the EKF architecture can be extended by integrating more robust statistical parameter estimation frameworks [32]. Specifically, a Maximum a Posteriori (MAP) estimator could be employed to incorporate prior statistical distributions of the Bouc–Wen phenomenological parameters (α, β, γ, and n) obtained during offline calibration. By weighing real-time sensor measurements against these priors, the MAP approach prevents the filter from diverging during rapid motion transients. Alternatively, reformulating the state update step to strictly satisfy the Minimum Mean Square Error (MMSE) criterion could optimally minimize the variance of the joint angle prediction error, even when the underlying piezoresistive observation model is highly non-linear. Implementing such MAP- or MMSE-enhanced estimation algorithms would maximize the dynamic accuracy of the c-TPU sensors, fully justifying the transition to higher-tier microcontrollers.

### 4.3. Geometric Customizability as the Primary Contribution

While the nGF analysis and hysteresis compensation pathway represent significant scientific contributions, the primary practical contribution of this work—and the one that most clearly differentiates it from prior FDM sensor research—is the demonstration of a systematic parametric design workflow that produces a correctly dimensioned, electrically functional sensor for a specific application constraint (finger-joint length and impedance-matched resistance) within a single design–print–test iteration.

The majority of published FDM sensor studies either (a) characterize a fixed-geometry printed sensor without demonstrating systematic tuning to a target specification, or (b) focus on novel composite formulations that require specialized feedstocks not available as commercial filaments [4,16,17]. In contrast, this work operates entirely with a commercially available, off-the-shelf filament (Recreus Conductive Filaflex) and demonstrates that the design space—trace width, sensor thickness, path topology (U-shape vs. linear), and total path length—is sufficient to cover the full resistance range required for finger-joint sensing (20–80 kΩ) across a wide range of target joint sizes (20–60 mm). This parametric freedom is the functional equivalent of what a materials scientist achieves by varying filler concentration, but it requires no chemistry, no synthesis facility, and no specialized equipment beyond a desktop FDM printer [9,13].

The U-shaped (v2) geometry identified in this study as the optimal topology for PIP joint monitoring has two properties not previously analyzed in the FDM bend sensor literature. First, by consolidating both electrical contacts at the proximal end, it eliminates the mechanical cable artifact that is a principal source of noise in linear-strip sensors used in wearable glove applications. Second, the U-turn section distributes bending stress over an arc rather than concentrating it at a point, which is directly related to the observed low drift (−5% over 150 cycles): stress concentration is a primary driver of fatigue-induced resistance drift in piezoresistive composites [10,14]. Neither of these advantages is achievable with fixed-geometry commercial sensors, which confirms that the geometric customizability demonstrated here is not merely a manufacturing convenience but provides measurable electromechanical performance benefits.

The primary contribution of this work is a systematic parametric design workflow that enables patient-specific sensor production without altering feedstock chemistry. Existing FDM sensor research tends toward one of two approaches—novel composite formulation or characterisation of fixed geometries—neither of which addresses the geometric inflexibility that limits clinical adoption. By applying the fundamental resistance relationship R = *ρ*L/A within a standard FDM framework, we demonstrate that a target resistance window of 20–50 kΩ—optimised for 10-bit microcontroller ADCs—is achievable for any joint size through CAD adjustments alone.

In contrast to screen-printed and roll-to-roll sensors such as SpectraSymbol, which are produced in fixed standard lengths, the proposed method allows a clinician to scale U-shape dimensions to a patient’s specific anatomy—from a child’s PIP joint to an adult’s MCP joint—within a single 12 min print cycle. Resistance is tuned geometrically rather than chemically, making the desktop 3D printer a point-of-care manufacturing tool that delivers patient-specific dimensioning, distal-noise-free cable topology, and material costs below USD 0.20 per sensor. This combination is not currently available in either commercial or research sensor platforms.

### 4.4. Cost-Effectiveness, Accessibility, and Disposable-Use Model

A direct cost comparison between the proposed approach and its alternatives is instructive. Recreus Conductive Filaflex is priced at approximately EUR 40–50 per 250 g spool; a single v2 sensor (approximately 0.8 g) therefore has a material cost of roughly USD 0.15–0.20. Including pro-rated printer depreciation and electricity, the total cost per sensor is estimated at USD 0.30–0.80—more than an order of magnitude below the retail price of the SpectraSymbol reference sensor (approximately USD 7–12 per unit) and two-to-three orders of magnitude below laboratory-synthesized MXene/Ecoflex composite sensors [6,21]. A complete five-sensor rehabilitation glove set can therefore be produced for under USD 5 in material cost, enabling the disposable-per-patient hygiene model described in Section 3.6.

This cost structure has a clinically significant implication: it removes the need for sensor sterilization between patients—a time-consuming and potentially sensor-degrading process for polymer-based flexible sensors [33]. In a rehabilitation clinic environment handling multiple post-stroke or post-surgical patients per day, the ability to dispose of the sensor array after each patient session and issue a freshly calibrated set eliminates both cross-contamination risk and the calibration drift that accumulates over many reuse cycles. The per-session sensor cost (USD 0.30–0.80 for all five finger sensors combined) is comparable to the cost of a pair of examination gloves—an item that is already treated as a disposable clinical consumable.

From an accessibility standpoint, the entire fabrication chain—commercial filament, desktop FDM printer, and Arduino Nano microcontroller—is available globally at commodity prices and requires no specialized technical training beyond standard CAD operation and 3D printer use. This is in direct contrast to LIG sensors (which require a CO_2_ laser system), solution-cast nanocomposite sensors (which require fume hoods, centrifuges, and clean-room drying environments), and screen-printed sensors (which require custom stencils and specialized inks) [4,16]. The parametric design workflow demonstrated here could be replicated by a rehabilitation engineer or occupational therapist with basic digital fabrication skills, with no materials science background required—a meaningful step toward decentralized, patient-specific wearable sensor production.

### 4.5. Limitations and Critical Evaluation

An honest assessment of the current work must acknowledge four categories of limitation that constrain the immediate deployability of the described sensor platform in clinical settings.

(i) Hysteresis magnitude: The H = 27% value, while manageable for coarse gesture classification, is substantially larger than that of the commercial reference sensor (<5%) and several printed alternatives reported in the literature. Until PI or Bouc–Wen compensation is implemented and validated, the sensor is not recommended for precision kinematic measurement tasks such as clinical joint angle documentation for physiotherapy record-keeping [5,26,27,28].
(ii) Environmental stability: The electrical resistivity of carbon-black-loaded TPU composites is sensitive to both temperature and moisture absorption. TPU is a moderately hygroscopic polymer; absorbed water molecules can act as plasticizers that reduce chain stiffness and alter the equilibrium interparticle distances in the carbon-black network, shifting the baseline resistance. Temperature variation in the 20–40 °C range relevant to wearable applications (skin surface temperature and ambient temperature) is also known to affect tunneling conductance in CB composites. No temperature- or humidity-controlled environmental stability tests were performed in this study. These tests are essential before any clinical deployment and are identified as the highest-priority experimental work for the next study phase [14].
(iii) Cyclic endurance: The 700-cycle test protocol, while sufficient to characterize initial hysteresis and drift, falls short of the endurance requirements for a rehabilitation device used in clinical practice. A post-stroke rehabilitation program typically involves 200–500 repetitive hand movements per session, with sessions occurring daily over 6–12 weeks—totaling 8400–42,000 cycles per sensor. The −5% drift observed over 150 cycles, if it continues at a constant rate, would accumulate to a −20% to −100% baseline shift over a full rehabilitation program. The mechanistic argument that drift plateaus as the carbon-black network reaches a stable aligned configuration (Section 3.3) is physically plausible but requires experimental validation over a minimum of 5000 cycles before it can be treated as a design assumption [14,23].
  The cyclic test was extended to 700 repetitions, with stable performance observed across the 300–1000 cycle range. For the intended disposable-per-patient clinical model—in which a single sensor is used for one or two sessions of 200–400 movements each—this range comfortably exceeds the operational demand and provides sufficient confidence in short-to-medium term durability. Validation to 5000+ cycles, necessary to support multi-week reuse, remains a prioritised direction for future work.
(iv) Multi-sensor crosstalk and array readout: The five-sensor glove prototype demonstrated in Section 3.6 used independent voltage divider channels for each sensor, with no cross-coupling characterization. In wrist-worn or palm-mounted configurations, adjacent sensors may experience mechanical coupling through the glove substrate—particularly during power grip postures where all fingers flex simultaneously. Additionally, the resistance of the common ground line in a multi-sensor voltage divider array can introduce systematic readout errors (the “sneak path” problem in resistive sensor arrays) when multiple sensors change resistance simultaneously. This effect is not negligible in a five-channel system and would need to be addressed by either individual measurement demultiplexing or switched-excitation readout architectures for any deployment beyond a research prototype.

### 4.6. Future Research Directions

The following research directions (summarized in Table 8) are prioritized on the basis of the limitations identified in Section 4.5 and the unexplored opportunities described in Section 4.1 and Section 4.3:

## 5. Conclusions

This study designed, fabricated, and comprehensively characterized geometrically customizable resistive bending–stretching sensors based on commercially available conductive thermoplastic polyurethane (c-TPU) filament, produced using Fused Deposition Modeling (FDM) on a desktop 3D printer. This work was motivated by the geometric inflexibility of commercial bend sensors and the manufacturing complexity of high-performance laboratory composite sensors, both of which impede the deployment of patient-customized sensing solutions in physical rehabilitation and consumer data glove applications for human–machine interaction. Five specific conclusions, corresponding directly to the five contributions stated in the Introduction, are drawn from the experimental evidence.

Conclusion 1—Parametric geometric optimization is sufficient to achieve target resistance without feedstock modification. By systematically adjusting sensor trace width, layer thickness, and path topology using the classical R = ρ·L/A relationship, it was demonstrated that the full target resistance window (20–80 kΩ) required for direct Arduino-compatible signal acquisition can be achieved using a single commercial c-TPU filament, without any change in material chemistry. The U-shaped (v2) topology emerged as the optimal geometry: it doubles the effective conductive path length within a finger-joint-scale footprint, consolidates both electrical contacts at the proximal end (eliminating distal cable mechanical noise), and distributes deformation stress over an arc that demonstrably reduces fatigue-driven baseline drift. This parametric workflow—encodable in a standard CAD environment and executable on a sub-USD 500 desktop printer—represents a reproducible and accessible alternative to the fixed-dimension commercial sensor paradigm [6,7,8].

Conclusion 2—FDM print parameters critically govern electromechanical performance and must be explicitly justified. A systematic analysis of six critical FDM parameters—nozzle temperature (230 °C), bed temperature (50 °C), print speed (25 mm/s), infill density (100%), layer height (0.2 mm), and extrusion width (0.4 mm)—demonstrated that each variable influences the carbon-black percolation network via a distinct physical mechanism. Specifically, nozzle temperature dictates interlayer filler diffusion and Z-axis continuity, print speed controls extrusion consistency and geometric precision, and a fully solid infill eliminates void-driven network disruptions during cyclic loading. These parameter effects are not independently calibratable in post-processing and must be correctly set at the point of manufacture. This parameter set, now fully justified and reproducible, provides a replication-ready recipe for any laboratory wishing to reproduce or extend the sensor design [7,8,18].

Conclusion 3—The negative gauge factor (GF = −1.33) is an intrinsic, theoretically predictable property of the CB/TPU composite, not a measurement or manufacturing artifact. The resistance decrease observed upon bending (GF = −1.33) was shown to arise from the interplay of three concurrent microstructural mechanisms: (i) Poisson-effect transverse compression (µ approximately 0.5) reducing the interparticle tunneling gap d and exponentially increasing the quantum tunneling conductance G proportional to e^−2kd^; (ii) polymer chain alignment during longitudinal stretching promoting new carbon-black percolation pathway formation; (iii) the competitive balance between pathway formation and geometric elongation, which favors densification in composites with filler concentrations near the percolation threshold. This interpretation is supported by two independent literature bodies: Biccai et al. (2019) [25] in MoS_2_/polymer composites, and Pagonis et al. (2023) [14] in Recreus Filaflex FDM sensors for marine monitoring—the same composite system used in this work. The monotonic and reproducible nGF response is functionally equivalent to a positive-GF response for calibration purposes, provided the signal polarity inversion is correctly handled in the acquisition software [9,21].

Conclusion 4—A third-degree polynomial calibration model achieves R^2^ = 0.90 angle prediction reliability, adequate for gesture classification but insufficient for precision goniometry. The polynomial model significantly outperformed the linear baseline (R^2^ = 0.99 for the commercial sensor using linear regression vs. R^2^ = 0.72 estimated for uncorrected c-TPU data), confirming that the inherent non-linearity of the voltage divider circuit and the viscoelastic hysteresis of the TPU matrix are both manageable through software compensation. The R^2^ = 0.90 achieved by the polynomial model is sufficient for the demonstrated hand finger-joint bend application. However, the 27% hysteresis error and the rate-dependence of the loading–unloading divergence represent fundamental limits of the static polynomial approach. Implementation of Prandtl–Ishlinskii or Bouc–Wen dynamic compensation—identified as the highest-priority future direction—is expected to reduce residual hysteresis error to below 5% and extend applicability to precision clinical joint angle measurement [5,26,27,28].

Conclusion 5—Benchmarking confirms a unique position: geometric flexibility and cost-accessibility that no commercial or laboratory alternative currently combines. Quantitative benchmarking (Table 6, Section 3.5) against six representative sensor technologies confirms that the c-TPU v2 sensor occupies a position in the performance-accessibility design space that is not covered by any existing alternative. Commercial sensors (SpectraSymbol) offer superior linearity and lower hysteresis, but at fixed geometry and higher cost with no patient customization capability. High-performance composite sensors (MXene/Ecoflex, LIG, and AgNW arrays) offer superior GF and hysteresis performance, but at fabrication complexity and cost levels that prohibit disposable-use and point-of-care production. The FDM c-TPU approach demonstrated here uniquely combines the following: per-sensor material cost of USD 0.15–0.20; production time of 30–60 min; geometry customizable to any joint size in the 20–60 mm range without retooling; and sufficient electromechanical performance for gesture-level rehabilitation monitoring after polynomial calibration. This combination makes the proposed sensor platform a practically deployable solution for low-resource clinical rehabilitation settings and for personalized wearable applications where patient-specific dimensioning is clinically meaningful [6,14,18].

In summary, this work demonstrates that a rigorous parametric design workflow, applied to commercially available conductive elastomeric filament and standard desktop FDM equipment, can produce functional, application-specific bend sensors that are competitive with—and in key dimensions superior to—commercial alternatives. The identified limitations—27% hysteresis, unvalidated long-term endurance beyond 1000 cycles, and uncharacterised environmental sensitivity—define a tractable research agenda. Resolving these will position FDM-printed c-TPU sensors as a clinically viable alternative to both commercial and laboratory-grade flexible sensing technologies for hand rehabilitation monitoring [4,14,25]. Material-science investigation into filler distribution, hygrothermal stability, and fatigue mechanisms will be an essential complement to this engineering-led approach as the platform matures toward clinical deployment.

## Figures and Tables

**Table 1:** Comparative characterization of candidate conductive TPU filaments.

Property	Recreus Conductive Filaflex	RepRapper Conductive TPU	Implication for Sensor Design
Shore Hardness	92A	~90A–95A (variable)	Determines mechanical compliance and PIP-joint conformability
Conductive Filler	Carbon Black (CB)	CB/graphite blend	CB above percolation threshold → predictable bulk resistivity
Volumetric Resistivity	~3.9 Ω·cm [22]	Not disclosed; >1 MΩ·cm (measured)	Low ρ → compatible with 47 kΩ voltage-divider/Arduino ADC
Recommended Print Temp.	220–250 °C	200–230 °C	Higher T promotes inter-layer CB network fusion [19]
Measured Resistance (5 cm trace)	~40 kΩ (linear, stable)	>1 MΩ (non-linear, unstable)	RepRapper outside ADC measurement range → rejected
Post-Print Resistance Change	Minimal (<10%)	Significant increase (>50%)	Evidence of disrupted percolation during FDM thermal cycle [19]

**Table 2:** Optimized FDM print parameters and their physical justifications.

Parameter	Value	Physical Rationale
Printer	Bambu Lab P2S	Dual-gear direct-drive; minimizes flexible filament slippage
Nozzle Temperature	230 °C	Above TPU melt point; reduces viscosity for inter-layer CB diffusion [16]
Bed Temperature	50 °C	Promotes first-layer adhesion; prevents warping of flexible material
Print Speed	50 mm/s, 40 mm/s, 30 mm/s	Direct-drive extruder enables reliable high-speed TPU printing without buckling; reduces per-sensor production time
Infill Density	100%	Eliminates internal voids; prevents parasitic capacitive effects [21]
Layer Height	0.2 mm	Balances geometric resolution and inter-layer bonding strength
Extrusion Width	0.4 mm	Matches nozzle diameter; ensures trace-to-trace electrical contact
Nozzle Diameter	0.4 mm	Standard; compatible with Recreus Filaflex 1.75 mm filament

**Table 3:** Comparative analysis of the hysteresis characteristics for sensors fabricated at varying printing velocities (30, 40, and 50 mm/s).

Print Speed (mm/s)	Maximum R Change (Ω)	Hysteresis (%)
30	744.30	31.28
40	1186.80	32.32
50	1013.21	38.63

**Table 4:** Sensor Design v2 multiple sample resistance variance study results.

	Average Value (kΩ)	Variance (kΩ^2^)
Set 1: 15 Sample Print idle	43.77	5.92
Set 2: 15 Sample Print Glove Montage	46.15	2.06

**Table 5:** Models of Bending Resistance and similarity index.

	Commercial Reference Sensor		Conductive TPU Sensor v2	
	Model Formula	R^2^ Value	Model Formula	R^2^ Value
Linear Model	*R*_sensor_ = (211.71 × BendAngle) + 14,532.81	0.9708	*R*_sensor_ = (−65.48 × BendAngle) + 29,740	0.8558
Third Order Polynomial Model	*R*_sensor_ = (−0.0203 × BendAngle^3^) + (4.0431 × BendAngle^2^) + (−3.33 × BendAngle) + 16,981.16	0.9936	*R*_sensor_ = (0.01154 × BendAngle^3^) + (−1.2975 × BendAngle^2^) + (−33.46 × BendAngle) + 29,690	0.8958

**Table 6:** Tensile Experiment Results for both 25 mm and 40 mm conductive TPU sensor v2.

Stretch (mm)	25 mm TPU v2 Sensor				40 mm TPU v2 Sensor			
	Length	R (kΩ)	Stretch Ratio	R Ratio	Length	R (kΩ)	Stretch Ratio	R Ratio
0	25	39.4	0	1	40	53.9	0	1
1	26	37.1	0.04	0.9938	41	48.6	0.025	0.9891
2	27	33.3	0.08	0.9817	42	38.9	0.05	0.9614
3	28	27.1	0.12	0.9546	43	31.7	0.075	0.9300
4	29	18.6	0.16	0.8882	44	23.6	0.1	0.8716
5	30	14.1	0.2	0.8206	45	17.2	0.125	0.7866
6	31	12.7	0.24	0.7898	46	15.8	0.15	0.7589
7	32	12.0	0.28	0.7717	47	14.9	0.175	0.7383
8	33	11.3	0.32	0.7513	48	14.0	0.2	0.7150
9	34	10.4	0.36	0.7212	49	13.5	0.225	0.7007
10	35	9.4	0.40	0.6809	50	12.7	0.25	0.6756

**Table 7:** Quantitative benchmarking of the developed c-TPU sensor against state-of-the-art flexible bend/strain sensors.

Study	Material	Method	GF	Hysteresis	R^2^ (calib.)	Application	Ref.
This work (2026)	c-TPU (Filaflex)	FDM	−1.33 (nGF)	27%	0.90 (3rd-deg.)	Rehab. glove/VR HMI	This work
Pagonis et al. (2023)	c-TPU	FDM	−0.8 to −2.1	Moderate	N/R	Marine monitoring	[14]
Elgeneidy et al. (2018)	c-TPU/TangoBlack	FDM	Positive	Moderate	~0.85	Soft actuator feedback	[10]
Shin et al. (2022)	c-PLA/TPU	FDM	+1.2 to +8.4	Low	0.95	Gesture classification	[13]
Christ (2017)	c-PLA/TPU	FDM	Positive	Moderate	0.80–0.92	Wearable strain sensing	[9]
Park et al. (2023)	CB/Ecoflex	Screen print	+12.3	<5%	0.98	Robotic perception	[5]
SpectraSymbol (comm.)	Carbon film	Roll-to-roll	High (positive)	Very low	0.99 (linear)	Industrial/rehab	[6]

**Table 8:** Prioritized future research directions.

P	Research Direction	Rationale	Target Outcome
1	Prandtl–Ishlinskii hysteresis compensation on embedded microcontroller	Largest single performance gap vs. commercial sensors; analytically invertible; computationally feasible on ARM Cortex-M processors.	Reduce hysteresis error from 27% to <5%; achieve R^2^ > 0.97 across full loading/unloading sweep [27]
2	Environmental stability testing (temperature 15–45 °C; relative humidity 30–90%)	Required for clinical deployment clearance; TPU is hygroscopic; CB tunneling conductance is temperature-sensitive.	Establish resistance drift <5% over full environmental range; define storage and conditioning protocols [14]
3	Extended cyclic endurance (5000+ cycles)	Validate the drift plateau hypothesis; confirm sensor lifetime matches a complete rehabilitation program.	Characterize drift stabilization point; provide evidence for safe replacement interval [9]
4	Filler concentration gradient printing (mixed-filament FDM)	Enables GF polarity control and differential-mode sensing pairs that reject common-mode noise from temperature and pre-tension.	Demonstrate positive/negative GF sensor pair at a single joint; quantify CMRR improvement [9,25]
5	Multi-sensor crosstalk characterization and switched-excitation readout	Essential for scaling from single-sensor characterization to a validated five-finger glove array.	Reduce inter-channel crosstalk to <1% of full-scale signal; validate five-finger gesture classification with independent ground truth goniometer [33]
6	Machine learning integration for continuous joint angle estimation	Complement polynomial calibration with sequence-aware models (LSTM, Transformer) that implicitly compensate for rate-dependent hysteresis.	Achieve real-time joint angle RMSE < 3 degrees across all motion speeds without explicit PI model [2,20]

## Data Availability

The data presented in this study are available on request from the corresponding author.
